# Glycogen synthase kinases in model and crop plants – From negative regulators of brassinosteroid signaling to multifaceted hubs of various signaling pathways and modulators of plant reproduction and yield

**DOI:** 10.3389/fpls.2022.939487

**Published:** 2022-07-15

**Authors:** Karolina Zolkiewicz, Damian Gruszka

**Affiliations:** Faculty of Natural Sciences, Institute of Biology, Biotechnology and Environmental Protection, University of Silesia, Katowice, Poland

**Keywords:** brassinosteroids, crosstalk, glycogen synthase kinases, plant reproduction, plant yield, signaling, stress response

## Abstract

Glycogen synthase kinases, also known as SHAGGY-like Kinases (GSKs/SKs), are highly conserved serine/threonine protein kinases present both in animals and plants. Plant genomes contain multiple homologs of the *GSK3* genes which participate in various biological processes. Plant GSKs/SKs, and their best known representative in *Arabidopsis thaliana –* Brassinosteroid Insentisive2 (BIN2/SK21) in particular, were first identified as components of the brassinosteroid (BR) signaling pathway. As phytohormones, BRs regulate a wide range of physiological processes in plants – from germination, cell division, elongation and differentiation to leaf senescence, and response to environmental stresses. The GSKs/SKs proteins belong to a group of several highly conserved components of the BR signaling which evolved early during evolution of this molecular relay. However, recent reports indicated that the GSKs/SKs proteins are also implicated in signaling pathways of other phytohormones and stress-response processes. As a consequence, the GSKs/SKs proteins became hubs of various signaling pathways and modulators of plant development and reproduction. Thus, it is very important to understand molecular mechanisms regulating activity of the GSKs/SKs proteins, but also to get insights into role of the GSKs/SKs proteins in modulation of stability and activity of various substrate proteins which participate in the numerous signaling pathways. Although elucidation of these aspects is still in progress, this review presents a comprehensive and detailed description of these processes and their implications for regulation of development, stress response, and reproduction of model and crop species. The GSKs/SKs proteins and their activity are modulated through phosphorylation and de-phosphorylation reactions which are regulated by various proteins. Importantly, both phosphorylations and de-phosphorylations may have positive and negative effects on the activity of the GSKs/SKs proteins. Additionally, the activity of the GSKs/SKs proteins is positively regulated by reactive oxygen species, whereas it is negatively regulated through ubiquitylation, deacetylation, and nitric oxide-mediated nitrosylation. On the other hand, the GSKs/SKs proteins interact with proteins representing various signaling pathways, and on the basis of the complicated network of interactions the GSKs/SKs proteins differentially regulate various physiological, developmental, stress response, and yield-related processes.

## Introduction

**FIGURE 1:**
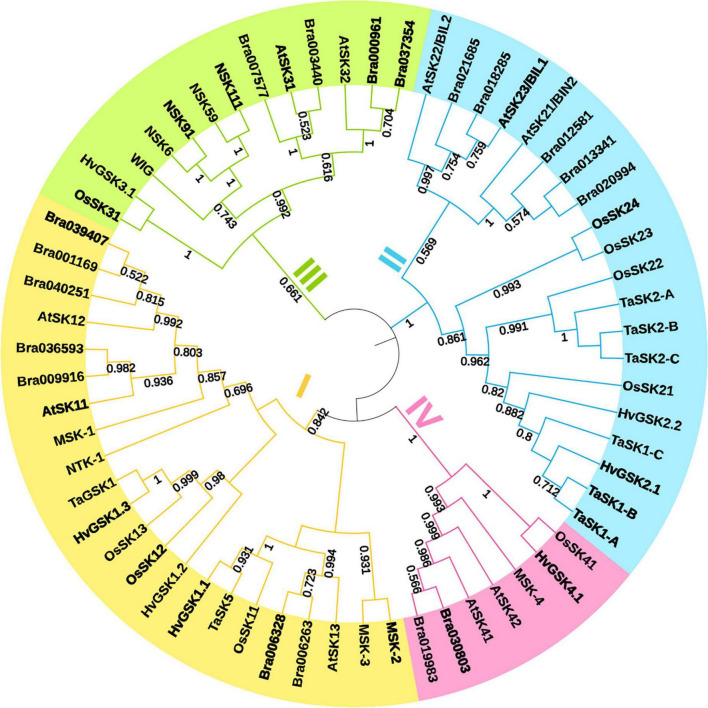
Maximum-likelihood phylogenetic tree representing the glycogen synthase kinases (GSK) family proteins from *Arabidopsis thaliana* (At), *Brassica rapa* (Bra), *Hordeum vulgare* (Hv), *Medicago sativa* (Ms), *Nicotiana tabacum* (Nt), *Oryza sativa* (Os), and *Triticum aestivum* (Ta). All kinases were clustered into four clades: I (yellow), II (blue), III (green) and IV (pink). Phylogenetic analysis was performed using MEGA X software. Bootstrap support values over 50%, calculated from 1000 replicates, are shown under branches. Visualization of the phylogenetic tree was designed by the iTOL v6 tool.

Glycogen synthase kinases, also known as SHAGGY-like Kinases (GSKs/SKs), are highly conserved serine/threonine protein kinases present both in animals and plants. In contrast to animal genomes which contain only few genes encoding these enzymes, plant genomes contain multiple homologs of the *GSK3* genes which participate in various biological processes (Yoo et al., 2006; Saidi et al., 2012; Youn and Kim, 2015; Li et al., 2021). However, similar to animal homolog**s,** generally these kinases are active under normal growth conditions, but are inactivated upon perception of various developmental or environmental signals (Mao et al., 2021). Plant *GSK* gene lineage emerged early in the evolution of plants, and their diversification process began before origin of seed plants (Yoo et al., 2006). It was suggested that the *GSK* genes may have played a crucial role in development of stress-tolerance mechanisms during adaptation of plants to life outside the aqueous environment. In plants, GSKs/SKs constitute a family of proteins which is divided into several subfamilies. Most probably, the diversification of the *GSK* genes has occurred more than once during evolution of the land plants (Richard et al., 2005; Yoo et al., 2006; Saidi et al., 2012). As far as dicot and monocot model species are concerned, there are ten genes encoding the GSK3-like kinases in Arabidopsis genome and nine genes encoding these kinases in the rice genome. Thus, it was postulated that the GSK3s family members may have performed diverse biological functions after the gene duplication event which occurred during plant evolution. The duplication of the *GSK* genes is prevalent in Arabidopsis and rice and occurred also in basal angiosperms (Yoo et al., 2006; Qi et al., 2013). Structure of the *GSK* genes in Arabidopsis and rice is highly conserved (Yoo et al., 2006). Apart from Arabidopsis and rice, genes encoding the GSK3-like proteins have been also identified in several important crop species, such as wheat (*Triticum aestivum*) (Chen et al., 2003; Cheng et al., 2020; Zhang et al., 2022), barley (*Hordeum vulgare*) (Groszyk et al., 2018; Kloc et al., 2020), maize (*Zea mays*) (Hou L. et al., 2022; Wang et al., 2022), sorghum (*Sorghum bicolor*) (Hirano et al., 2017), cotton (*Gossypium hirsutum*) (Wang et al., 2018a), soybean (*Glycine max*) (Wang et al., 2018b; He et al., 2021), potato (*Solanum tuberosum*) (Huang et al., 2021), and pepper (*Capsicum annuum*) (Qiu et al., 2018). Although, our current understanding of their functions in these crop species is far from complete, the reports indicate that roles of these GSK proteins in plant development and stress responses are similar to those in Arabidopsis and rice (Li et al., 2021). However, research on some of these crop species provided an important information which was not available from studies conducted in the model species, including roles of the GSK proteins in regulation of plant-microbe interactions (He et al., 2021). Recently, genome-wide identification and expression analysis of the *GSK* gene family in the important crop species, wheat (*Triticum aestivum*), have been described for the first time. In this study, 22 *GSK* genes in the wheat genome were identified. On the basis of homology analysis and phylogenetic relationships with the *GSK* genes from Arabidopsis, rice and barley, the *GSK* genes of wheat were clustered into four subfamilies. Interestingly, the *GSK* genes clustered in the same subfamily share similar exon/intron organization, and the encoded proteins show similar motif structures. Analysis of promoter sequences of the wheat *GSK* genes indicated that they are involved in plant growth and development, as well as in the hormone, light and abiotic-stress signaling pathways. In accordance with these results, the *GSK* genes of wheat exhibited preferential expression in specific tissues and differential expression patterns under the abiotic stress conditions (mostly drought and salinity). Some of the *GSK* genes in wheat were abundantly expressed in spikes and grains at a specific developmental stage (Zhang et al., 2022). These results may suggest a regulatory role in reproductive development and potential influence on yield. In barley, seven transcriptionally active *GSK* genes assigned to four groups have been identified. The encoded proteins showed high level of conservation of functional domains and motifs. The barley *GSK* genes are ubiquitously expressed in various organs and at different developmental stages. Interestingly, the spatial transcriptional patterns of homologous *GSK* genes from the various groups indicated a shift in organ-specific expression in Arabidopsis and barley. Particularly, the group four of the *GSK* genes which encode proteins preferentially involved in the osmotic stress response became more biologically relevant in barley which is adapted to more dry environmental conditions than Arabidopsis. Therefore, it was suggested that a diversification of biological roles of individual *GSKs* in these plant species occurred (Groszyk et al., 2018). Recently, two GSK homologs in maize have been identified and reported to be expressed ubiquitously in various tissues. However, one of the homologs (*ZmGSK2*) seems to be the major regulator of developmental processes in this species (Hou L. et al., 2022). A study of the GSK3 homolog LjSK1 in *Lotus japonicus* allowed for identification of regulatory mechanism which modulates activity of this kinase. Previous study indicated that this kinase is involved in nodule development. Interestingly, removal of a 98 residue-long N-terminal fragment of this protein resulted in almost two-fold increase in its catalytic efficiency (Solovou et al., 2021). Classification, nomenclature and phylogenetic relationship between various representatives of this protein family are presented in Figure 1 and in Supplementary Table 1.

## Role of the glycogen synthase kinase 3 proteins in the brassinosteroid signaling

**FIGURE 2:**
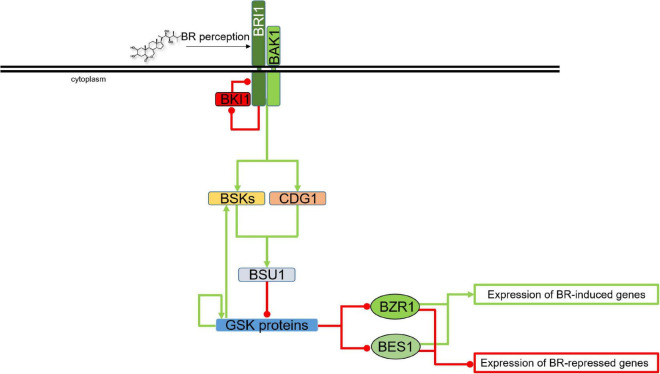
Role of the GSK proteins in regulation of the BR signaling. Green arrows indicate stimulation, whereas red lines with the bullet points represent inhibition. Transcription factors are shown as ovals, other proteins are depicted as rectangles. Double black line represents plasma membrane. Detailed description is given in the text. BRI1, Brassinosteroid-Insensitive1; BAK1, BRI1-Associated receptor Kinase1; BKI1, BRI1 Kinase Inhibitor1; BSKs, BR-Signaling Kinases; CDG1, Constitutive Differential Growth1; BSU1, bri1 suppressor1 phosphatase; BZR1, Brassinazole-resistant1; BES1, bri1-EMS suppressor1.

Plant GSKs/SKs, and their best known representative in *Arabidopsis thaliana –* Brassinosteroid Insentisive2 (BIN2/SK21) in particular, were first identified as components of brassinosteroid (BR) signaling pathway. A gain-of-function mutation of the *BIN2* gene results in BR insensitivity (Li and Nam, 2002). Importantly, BIN2 belongs to a group of several highly conserved components of the BR signaling which evolved early in the evolution of this molecular relay, and homologs of BIN2 were identified in Chlorophytes, Liverworts, Mosses, Lycophytes, Monocots and Eudicots. The canonical BR signaling pathway emerged relatively late during plant evolution. Therefore, it has been suggested that BIN2 (and some other components of the BR signaling) might have participated in other molecular pathways before gaining the BR-related functions (Kim and Russinova, 2020). Two closest homologs of the *BIN2* genes, BIN2-like1 (BIL1) and BIL2 were identified later on, they act redundantly with BIN2 as negative regulators of the BR signaling, and the triple mutant *bin2/bil1/bil2* showed phenotype which indicated a constitutive activation of the BR signaling and BR response (Vert and Chory, 2006; Yan et al., 2009; Kim and Russinova, 2020). Since the discovery of brassinosteroids (BR) in the 1970’s, these plant steroid hormones have been proven to be important regulators of various aspects of plant development. The last four decades of the research on BRs broadened our knowledge. However there is still much more to be uncovered. Many reports highlighted the importance of BRs in a wide range of physiological processes in plants – from seed germination, cell division, elongation and differentiation to leaf senescence and response to biotic and abiotic stresses (Anwar et al., 2018; Peres et al., 2019; Ackerman-Lavert and Savaldi-Goldstein, 2020). The BR signaling has been well elucidated and currently is one of the best described molecular relays in plants, particularly in the model species *Arabidopsis thaliana* (Planas-Riverola et al., 2019; Gruszka, 2020; Kim and Russinova, 2020). In further studies, the GSKs/SKs proteins were also found to be implicated in signaling pathways of other phytohormones and stress-response pathways (Li et al., 2021; Mao et al., 2021). However, in order to properly present role of these kinases as hubs of various signaling pathways, coordinating different metabolic and physiological processes, their primary role of master (negative) regulators of the BR signaling should be first described in order to provide a context for characterization of other roles and functions. Role of the GSKs/SKs proteins in regulation of the BR signaling is presented in Figure 2.

Briefly, when BRs are present, they are recognized by leucine-rich repeat receptor-like (LRR-RLK) protein Brassinosteroid-Insensitive1 (BRI1), along with its co-receptor BRI1-Associated receptor Kinase 1, also known as Somatic Embryogenesis Receptor-like Kinase3 (BAK1/SERK3) at the plasma membrane. In order to fully initiate the signaling cascade, BRI1 phosphorylates its inhibitor BRI1 Kinase Inhibitor1 (BKI1), what results in inactivation of BKI1, thus enabling formation of the functional BRI1-BAK1 receptor complex. The BR-dependent heterodimerization of BRI1 and BAK1 brings their cytoplasmic kinase domains in a proper orientation for transphosphorylation and initiation of the signaling cascade (Wang et al., 2014; Gruszka, 2020). The BR signal transduction is subsequently propagated through phosphorylating other proteins, including the BR-Signaling Kinases (BSKs), Constitutive Differential Growth1 (CDG1) protein, and finally bri1 suppressor1 phosphatase (BSU1), which inhibits the major negative regulator of the BR signaling, Brassinosteroid Insensitive 2 (BIN2), one of the best characterized members of the Shaggy/Glycogen Synthase Kinase protein family in Arabidopsis (Mao and Li, 2020). As the major negative regulator of the BR signaling, under low BR levels the BIN2 kinase is constitutively active and interacts with, phosphorylates, and as a consequence, deactivates two transcription factors Brassinazole-resistant1 (BZR1) and bri1-EMS suppressor1 (BES1), which are master regulators of the BR-dependent gene expression (He et al., 2002; Zhao et al., 2002; Vert and Chory, 2006; Gruszka, 2013; Figure 2). In the BZR1 and BES1 transcription factors ten and nine amino-acid residues, respectively, have been experimentally validated as the target positions of the BIN2-mediated phosphorylation (Gampala et al., 2007; Ryu et al., 2007, 2010). Generally, BIN2 phosphorylates serine and threonine residues in the conserved motif S/T-X-X-X-S/T in target proteins (Zhao et al., 2002). However, it is known that BIN2 recognizes various regulatory motifs in different protein substrates (Youn and Kim, 2015). Upon initiation of the BR signaling, and due to the BIN2/AtSK21 inactivation, the transcription factors BZR1 and BES1 are able to bind to promoters of multiple BR-responsive genes (Vert and Chory, 2006; Gruszka, 2018; Nolan et al., 2020).

Studies conducted in Arabidopsis indicated that out of ten members of the AtSK/GSK family, at least seven (AtSK11, 12, 13, 21, 22, 23, and 31) are involved in the BR signaling in this species (Kim et al., 2009; Yan et al., 2009; Rozhon et al., 2010; Saidi et al., 2012; Youn et al., 2013) what may suggest a significant functional redundancy. Interestingly, the BZR family of transcription factors contains six members which function redundantly in the BR signaling (Yin et al., 2005). Thus, it is likely that each of the seven members of the AtSK family interacts with each member of the BZR family with different specificities which influence their functions in regulation of developmental and physiological processes (Youn and Kim, 2015). However, knowledge about substrate specificities of the various AtSKs involved in the BR signaling and their interacting proteins is still limited (Gruszka, 2018). Moreover, despite similar function in the BR signaling regulation, the GSK3 proteins may play diverse roles in regulation of growth and development of Arabidopsis plants. It indicates that different AtSKs may have overlapping as well as specific functions (Youn et al., 2013; Gruszka, 2018). Although it is known that generally BIN2 is a negative regulator of the BR signaling, a recent report indicated that BIN2-mediated phosphorylation stabilizes one of the members of the above-mentioned BSK protein family, BSK3, to promote its interaction with the BR receptor BRI1 and the BSU1 phosphatase, what finally results in stimulation of the BR signaling (Ren et al., 2019). Thus, it was suggested that BIN2 plays a dual role in regulating the BR signaling: firstly through the enhancement of the BSK3 interactions (positive influence on the BR signaling), and secondly through inactivation of the BZR1 and BES1 transcription factors (negative impact on the BR signaling) (Mao et al., 2021). However, when BRs are absent, the signal transduction does not occur because of BKI1, which prevents the BRI1-BAK1 interaction. In the absence of phosphorylating cascade, BSU1 is not activated what results in the BIN2 kinase stabilization. Furthermore, BIN2 phosphorylates the BZR1 and BES1 transcription factors causing their inactivation and cytoplasmic retention, and consequently the BR-dependent regulation of gene expression does not occur (Gruszka, 2013). Importantly, it was reported in Arabidopsis that when the BRs are absent, the BIN2 kinase maintains its activity through autophosphorylation of Tyr^200^ residue. This is one of the mechanisms which suppress the BR signaling during the absence of BR (Li et al., 2001; Li and Jin, 2006; Ye et al., 2011; Figure 2). Updated and comprehensive descriptions of the BR signaling pathway in model and crop species have been recently published (Gruszka, 2018, 2020; Nolan et al., 2020).

In rice, several members of the OsSK family, including OsSK22/GSK2, function as regulators of the BR signaling by regulation of stability and activity of several transcription factors which are directly involved in the BR-dependent gene expression (Tong et al., 2012; Yang et al., 2016; Qiao et al., 2017; Xiao et al., 2017; Min et al., 2019; Gruszka, 2020). Thus, function of the GSK3 proteins as negative regulators of the BR signaling is generally conserved among dicots and monocots, however, it should be mentioned that several components of the BR signaling in rice do not have orthologs in Arabidopsis. This observation indicates that some parts of the BR signaling pathway may be specific for rice and other monocots (Zhang C. et al., 2014; Gruszka, 2020). Details of this phosphorylation-dependent regulation will be described below.

## Various mechanisms of the glycogen synthase kinase stability and activity regulation

**FIGURE 3:**
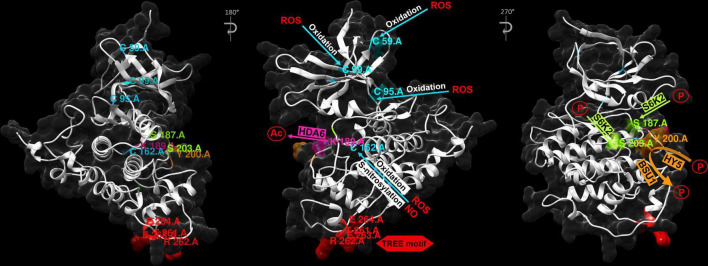
Three-dimensional structure of the AtSK21/BIN2 protein (UniProt Entry: Q39011) of Arabidopsis. Positions of the amino-acid residues: Cys^59^, Cys^95^, Cys^99^, Cys^162^, Ser^187^, Lys^189^, Tyr^200^, Ser^203^, Thr^261^, Arg^262^, Glu^263^, and Glu^264^ which are modified during regulation of the BIN2 protein activity are shown together with modifying factors. Ac, Acetyl group; P, Phosphate group. Visualization and analysis of the three-dimensional structure of the AtSK21/BIN2 protein was performed using the SWISS-MODEL database and UCSF Chimera program (version 1.10.1).

**FIGURE 4:**
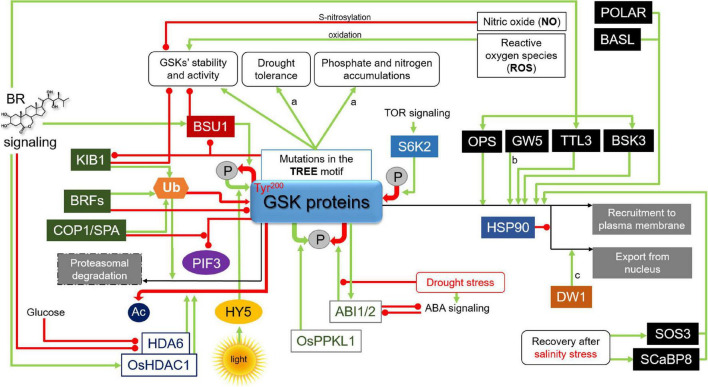
Mechanisms regulating stability and activity of the GSK proteins. Green arrows indicate stimulation, whereas red lines with the bullet points represent suppression. Solid ovals containing the letter “P” denote phosphorylation/dephosphorylation events, whereas “Ac” and “Ub” represent acetyl functional group and ubiquitylation, respectively. Positive and negative effects of these chemical modifications on the GSK proteins are represented by green and red arrows, respectively. Some of the regulatory mechanisms were reported in: a, Indian dwarf wheat; b, rice; c, sorghum. Detailed description is given in the text. KIB1, Kink Suppressed In bzr1-1D1; BRF, Brassinosteroid F-box; COP1/SPA, CONSTITUTIVELY PHOTOMORPHOGENIC1/SUPPRESSOR OF *phyA-105*; BSU1, bri1 suppressor1 phosphatase; PIF3, PHYTOCHROME INTERACTING FACTOR3; HY5, Elongated Hypocotyl5; OsPPKL1, Protein Phosphatase Kelch-Like1; ABI1/2, ABA-Insensitive1 and 2 protein phosphatases; S6K2, Ribosomal protein S6 kinase2; POLAR, Polar Localization During Asymmetric Division and Redistribution; BASL, Breaking of Asymmetry in the Stomatal Lineage; OPS, OCTOPUS; GW5, Grain Width5; TTL3, Tetratricopeptide Thioredoxin-like3; BSK3, BR-Signaling Kinase3; HSP90, Heat shock protein90; DW1, Dwarfing1; SOS3, Salt Overly Sensitive3; SCaBP8, SOS3-like Calcium Binding Protein8.

Importantly, in all the AtSK family members in Arabidopsis the kinase domains and C-terminal regions are highly conserved, whereas the N-terminal regions are usually divergent (Supplementary Figure 1). The C-terminal regions are mainly responsible for interactions with substrates. The divergent N-terminal regions influence their subcellular localization (Yoo et al., 2006; Youn and Kim, 2015). Stability of the BIN2 kinase is mainly regulated through its C-terminal TREE (Thr^261^-Arg^262^-Glu^263^-Glu^264^) motif which is a part of a surface-exposed α-helix (positions of amino acid residues which play important role in regulation of the BIN2 activity were shown in Supplementary Figure 1 alignment and in three-dimensional structure of BIN2 in Figure 3). The Glu263Lys amino-acid substitution in this motif, identified in the *bin2-1* mutant of Arabidopsis, proved to be a gain-of-function mutation. This mutation increases stability of BIN2 and renders it hyperactive, as it prevents the BSU1-mediated dephosphorylation and reduces proteasome-mediated degradation of the BIN2 kinase (Li and Nam, 2002; Peng et al., 2008; Mao and Li, 2020; Figure 4). Interestingly, among eight gain-of-function alleles of the *BIN2* gene, seven carried mutations in the TREE motif (Li et al., 2001; Choe et al., 2002; Li and Nam, 2002; Perez-Perez et al., 2002). Mutations in this motif were also identified in other GSK3 kinases of Arabidopsis, such as BIL1, BIL2, AtSK11, and AtSK12 (Li and Nam, 2002; Kim et al., 2009; Yan et al., 2009; Li et al., 2018), as well as in the OsGSK2 kinase of rice (Tong et al., 2012), the ZmGSK2 protein of maize (Hou L. et al., 2022), and the TaSG-D1 kinase of Indian dwarf wheat (*Triticum sphaerococcum*) (Cheng et al., 2020; Hughes, 2020). In the Indian dwarf wheat it was reported that this mutation has pleiotropic effects on unique, semi-spherical grain shape and plant architecture, and that origin of *T. sphaerococcum* in ancient India involved at least two independent mutations of *TaSG-D1* (Cheng et al., 2020; Hughes, 2020). The mutations identified in this motif in the dicot and monocot species caused similar gain-of-function effects which resulted from the higher stability of the homologous GSK3 proteins. These reports from the model and crop species indicate that the function of the TREE motif in regulation of the GSK3 stability is conserved during evolution of plants (Li et al., 2021). Moreover, another recent study in the Indian dwarf wheat indicated that mutations in the TREE motif resulted also in an enhanced drought tolerance and increased phosphate and nitrogen accumulations (Figure 4), and these physiological traits may be considered as adaptation to dry climate of India and Pakistan (Gupta et al., 2021).

The regulatory function of the TREE motif was further confirmed by identification of an F-box domain E3 ubiquitin ligase which regulates stability of the BIN2 kinase. It was reported that BIN2 is negatively regulated by the E3 ubiquitin ligase KIB1 (Kink Suppressed In bzr1-1D 1). KIB1 mediates the ubiquitylation-dependent degradation of BIN2 in proteasome in the presence of BRs, and this degradation is reduced by mutation in the TREE motif (Figure 4). Overexpression of KIB1 caused accumulation of unphosphorylated (active) transcription factor BZR1, and the level of BIN2 markedly decreased, demonstrating the correlation between KIB1 and BIN2 (Zhu et al., 2017). It is now known that KIB1 performs the BR-induced proteasomal degradation of BIN2, however, it is still not clarified how KIB1 interacts with other regulatory protein of the BR signaling (Li et al., 2021). It was shown that KIB1 is able to regulate the GSK3 kinase by two means. Firstly, KIB1 prevents BIN2 to interact with its substrate BZR1, which results in the elevated BZR1 accumulation. Secondly, the GSK3 kinase is subsequently directed to ubiquitination-mediated degradation. It is also interesting to mention that KIB1 is capable of interacting with other Arabidopsis GSK3 kinases belonging to the subgroup I and II. Moreover, it was reported that KIB1 and its three homologous proteins belonging to the same family act redundantly in the BR-dependent suppression of the GSK3 kinase activity. However, it is not yet known what is the exact mechanism of the BR-dependent activation of KIB1 (Zhu et al., 2017). More recently, it was also suggested that BIN2 kinase could be regulated by other E3 ligases. It was reported that the E3 ligase Brassinosteroid F-box 1 (BRF1) is able to interact with BIN2 and repress its function. Moreover, it was postulated that BRF1 and its homolog BRF2 are redundantly involved in regulation of the BIN2 stability (Figure 4). Accumulation of the BRF1 and BRF2 proteins is stimulated by BR (Jeong, 2020; Jeong et al., 2022). However, further studies are required to fully clarify this aspect. Interestingly, another E3 ubiquitin ligase complex CONSTITUTIVELY PHOTOMORPHOGENIC1/SUPPRESSOR OF *phyA-105* (COP1/SPA) was recently reported to inhibit interaction between BIN2 and its target transcription factor PHYTOCHROME INTERACTING FACTOR3 (PIF3) which mediates light signaling. However, the COP1/SPA complex does not affect the BIN2 stability (Ling et al., 2017). It indicates that although the KIB1, BRF, and COP1/SPA E3 ubiquitin ligases share the BIN2 kinase as a common target protein their regulatory impacts on this protein are different (Figure 4). It may result from alternative modifications of amino acid residues in BIN2 or different interacting proteins.

A common mechanism of regulation of protein activity is post-translational modification. Plant GSK3s are also regulated at the post-translational level by phosphorylation, acetylation, nitrosylation and ubiquitination. These post-translational molecular modifications and interactions between plant GSK3s and their regulatory proteins or substrates lead to changes in stability, activity and subcellular localizations of GSK3s (Mao and Li, 2020; Li et al., 2021). It is well known that autophosphorylation of Tyr^200^ residue of BIN2 is required for its kinase activity in the absence of BR (Ye et al., 2011). Activity of BIN2 is suppressed by dephosphorylation of this residue after BR perception and initiation of the BR signaling cascade (Li et al., 2001; Clouse, 2002; Perez-Perez et al., 2004; Li and Jin, 2006). The BR-dependent dephosphorylation of the Tyr^200^ residue is catalyzed by the aforementioned BSU1 phosphatase (Ye et al., 2011; Figures 3, 4).

Interestingly, it was reported that phosphorylation of Tyr^200^ is stimulated by Elongated Hypocotyl5 (HY5) (Figure 3), which functions as a positive regulator of photomorphogenesis downstream of multiple photoreceptors (Gangappa and Botto, 2016; Li and He, 2016; Li et al., 2021). It was recently reported that HY5, which is a basic domain/leucine zipper (bZIP) transcription factor involved in photomorphogenesis, has a positive effect on the BIN2 activity. Interestingly, HY5 does not influence expression of the *BIN2* gene. HY5 interacts with BIN2 through its C-terminal domain, promoting the BIN2-mediated phosphorylation and deactivation of the BZR1 transcription factor in the light-dependent manner. Moreover, it was also reported that HY5 is able to interact with the two closest homologs of BIN2 – BIL1 and BIL2. Positive regulation of BIN2 by HY5 results in promoting the BIN2 activity in order to inhibit hypocotyl elongation and promote photomorphogenesis. Accumulation of HY5 protein increases with light intensity which results in stimulation of the BIN2 activity (Figure 4). Interestingly, it was reported that the Leu^137^ residue of HY5 is important for the HY5-BIN2 interaction, however, this residue does not affect the transcriptional activity of HY5 (Li et al., 2020a). These results indicate that phosphorylation of the Tyr^200^ residue in BIN2 is an example of antagonistic interaction between the BR and light signaling pathways (Figure 4). It was recently reported that another photoreceptor, Cryptochrome1 (CRY1), which initiates blue-light signaling pathway, interacts with BIN2 and the BZR1 transcription factor to facilitate the BIN2-mediated inactivation of BZR1 in the light-dependent manner. This alternative mechanism also results in the inhibition of hypocotyl elongation (He et al., 2019).

Noteworthy, phosphorylation of the BIN2 kinase may lead to its inactivation, and this effect is dependent on position of phosphorylated residues. It was reported that BIN2 activity is also regulated by the target of rapamycin (TOR) signaling, which promotes plant growth under appropriate conditions, by regulating metabolic processes (Burkart and Brandizzi, 2021). The TOR signaling inactivates BIN2 through the Ribosomal protein S6 kinase2 (S6K2), as S6K2 is localized in the same compartment as the BIN2 kinase. It was reported that the BIN2 kinase is phosphorylated by S6K2 at the Ser^187^ and Ser^203^ residues (Figures 3, 4). Importantly, analysis of the BIN2 involvement in TOR signaling demonstrated that BIN2 negatively regulates photoautotrophic growth (Xiong et al., 2017; Fabregas and Fernie, 2021). However, it was recently reported that there is an alternative mechanism of interaction between the sugar metabolism and the BIN2 activity, and this regulation is dependent on the light/darkness conditions. It is known that sugar stimulates the BR signaling and hypocotyl elongation in the darkness. On the contrary, under the light conditions sugar represses the BR signaling, and consequently seedling growth. This inhibitory effect of sugar is independent of the TOR signaling (the pathway is currently unknown), but requires BIN2 which is stabilized by sugar under the light conditions. In this way, the light/darkness conditions-dependent interplay between the sugar metabolism and BR signaling allows for optimal adjustment of plant physiology and growth rate to the environmental conditions (Zhang Z. et al., 2021).

Interestingly, dephosphorylation of GSK3 does not always result in repression of its activity (as it is in the case of the BSU1 phosphatase effect). It was reported in rice that Protein Phosphatase Kelch-Like1 (OsPPKL1) directly interacts with the rice OsGSK3 kinase and dephosphorylates it. However, in contrast to the dephosphorylation of Arabidopsis BIN2 kinase, which results in the BIN2 degradation, in rice OsPPKL1 dephosphorylates and stabilizes the OsGSK3 protein in the cytoplasm (Figure 4). Thus, OsPPKL1 is a negative regulator of the BR signaling in rice by enhancing stability of OsGSK3 (Gao et al., 2019). Regulation of the OsGSK3 stability by the OsPPKL1-mediated dephosphorylation constitutes a significant difference in the BR signaling model between rice and Arabidopsis (Gruszka, 2020). It was suggested that different phosphorylation sites in BIN2 of Arabidopsis and OsGSK3 of rice may be responsible for the opposite effect on stabilization. However, further studies are needed to better understand the differences in regulatory mechanisms of the GSK3 orthologs between dicots and monocots and their respective involvement in plant physiology. Noteworthy, apart from the involvement in the OsGSK3 stability, OsPPKL1 phosphatase regulates grain length in rice. It seems that within the rice genome a neofunctionalization among homologous OsPPKL proteins occurred. There are three homologs of the OsPPKL proteins in the rice genome (Gao et al., 2019). Interestingly, two of these homologs, OsPPKL1 and OsPPKL3, play a negative role in the regulation of grain length, whereas OsPPKL2 has a positive effect on the grain length in rice (Zhang X. et al., 2012).

It is known that BIN2 is also dephosphorylated by the ABA-Insensitive1 and 2 protein phosphatases (ABI1 and 2) which play a role of negative regulators of the abscisic acid (ABA) signaling (Weiner et al., 2010). Activity of the ABI phosphatases is suppressed by ABA. The ABI1 and 2 phosphatases directly interact with BIN2 and dephosphorylate this kinase to inhibit its activity (Figure 4). Moreover, the GSK proteins are likely the major targets of the ABI phosphatases. As a consequence, the ABI1 and 2 phosphatases play a positive role in regulation of the BR signaling, and the BIN2 kinase constitutes a hub in a crosstalk between the BR and ABA signaling pathways (Gruszka, 2018; Wang et al., 2018c; Jiang et al., 2019). Interestingly, it was reported that the ABI1-mediated dephosphorylation of BIN2 is more efficient when ABI1 is phosphorylated, and the ABI1 phosphorylation may be performed by BIN2 in a form of feedback loop (Jiang et al., 2019; Figure 4). However, target residues in BIN2 which are dephosphorylated by the ABI1 and 2 phosphatases are still unknown (Li et al., 2021). Taken together, the differential pattern of phosphorylation and de-phosphorylation of the GSK proteins regulates the interplay between the BR and other signaling pathways which underlies balance between growth and stress response under constantly changing environmental conditions (Gruszka, 2018; Jiang et al., 2019). Indeed, it was recently suggested that the ABI1- and ABI2-mediated dephosphorylation of BIN2 is inhibited by drought stress which is known to activate the ABA signaling (Jiang et al., 2019; Lv and Li, 2020; Figure 4).

Another mechanism of the post-translational modification of the BIN2 activity is deacetylation. Importantly, acetylation of the Lys^189^ position, localized in a conserved phospho-binding pocket of GSKs (Figure 3), is vital for the BIN2 function. Moreover, mutation at this position induces a partial repression of the BIN2 kinase activity, including decrease in its autophosphorylation activity. It was reported that the histone deacetylase6 (HDA6) is able to interact with BIN2 and remove its acetyl functional group (Figure 3). This reaction results in repressing the BIN2 protein activity (Figure 4), therefore, it indicates the positive role of HDA6 in the BR signaling. However, it was reported that absence of BIN2 and its close homologs resulted in reduced impact of HDA6 on the BR signaling. Interestingly, the expression level of the *HDA6* gene was also decreased in triple *bin2-3/bil1/bil2* knockout mutant, revealing a feedback regulation of the histone deacetylase by BIN2 and its homologs. Moreover, transcription of the *HDA6* gene is suppressed by the BR signaling in a feedback manner. Noteworthy, it was also reported that glucose may influence the BIN2 acetylation status and the HDA6-mediated repression of the BIN2 activity may occur only under energy-limiting conditions (Figure 4), what provided a link between the acetylation-dependent regulation of BIN2 and cellular energy status (Hao et al., 2016). However, this aspect requires further investigation. Recently, it was reported in rice that histone deacetylase1 (OsHDAC1) directly interacts with and deacetylates OsGSK2, what results in suppressing activity of this kinase. However, in contrast to the BR-dependent repression of expression of the *HDA6* gene in Arabidopsis, expression of the *OsHDAC1* gene is significantly induced by BR (Figure 4). The OsHDAC1-mediated deacetylation of OsGSK2 attenuates interaction between OsGSK2 and its target transcription factor BRASSINAZOLE-RESISTANT 1 (OsBZR1) what results in the OsBZR1 accumulation. Apart from elucidation of this molecular mechanism, it was also indicated that it is associated with lateral root formation in rice. OsHDAC1 regulates the lateral root formation by deactivating OsGSK2, thereby preventing degradation of OsBZR1, a positive regulator of lateral root primordia development. It was also suggested that OsHDAC1 may become a target in breeding programs of rice, as it may improve resource uptake, and therefore may be of significant agronomic value. However, it is still unknown which histone acetylases perform acetylation of OsGSK2 and what is the exact regulatory mechanism of synergistic phosphorylation and acetylation of OsGSK2 (Hou J. et al., 2022). Generally, these reports indicate that the histone deacetylase-mediated regulation of the GSK3 activity is conserved among dicots and monocots, however, the mechanisms of BR-dependent regulation of expression of genes encoding the histone deacetylases are opposite between Arabidopsis and rice, what requires further research.

Activity of the BIN2 kinase may also be regulated by oxidation. Although the negative role of reactive oxygen species (ROS) has been known for a long time, involvement of these metabolic by-products in mechanisms of plant response to abiotic stress is still being uncovered. The interaction between ROS and hormone signaling pathways during drought, heat, cold, and salt stress reveals their importance in regulation of plant physiology under the stress conditions (Devireddy et al., 2021). Interestingly, ROS positively regulate the activity of BIN2 (Figure 4). It was demonstrated that the low level of oxygen within the cell reduced the ability of BIN2 to interact with its target transcription factor BES1, which indicates a crucial role of oxygen in the BIN2 activation. Under the hypoxia conditions, the lower level of BES1 in the cytoplasm was reported, despite of the presence of BIN2. Importantly, it was concluded that ROS regulate the BIN2 activity by oxidation of the following cysteine residues: Cys^59^, Cys^95^, Cys^99^ and Cys^162^ (Figure 3), which in oxidized state cause activation of the BIN2 kinase (Song et al., 2019). It was recently suggested that the ROS-mediated activation of the BIN2 kinase plays a role during plant response to the heat stress (Lv and Li, 2020). This conclusion is related with the recent reports describing role of BR in regulation of antioxidant homeostasis during reaction of semi-dwarf barley mutants to another stress factor – drought. In these reports it was concluded that proper progress of the BR biosynthesis and signaling processes is required for accumulation of the non-enzymatic antioxidants (ascorbate, glutathione, tocopherols) under optimal watering and drought conditions (Gruszka et al., 2018). Moreover, a significant role of BRs in regulation of the ascorbate peroxidase (HvAPX) and catalase (HvCAT) activities under optimal conditions and the HvAPX and superoxide dismutase (HvSOD) activities during physiological reactions to drought was also reported (Gruszka et al., 2020a). Taking the above aspects into account it may be concluded that the BR metabolism and redox homeostasis influence each other mutually.

Additionally, apart from the oxidation reactions, it was indicated that activity of BIN2 is inhibited by nitric oxide (NO) in the S-nitrosylation reaction (Wang et al., 2015; Figure 4). Nitric oxide is a molecule involved in several processes, including response to low metalloid and nutrient supply, and various abiotic stresses (Fancy et al., 2017; Buet et al., 2019). It was reported that NO inactivates BIN2 through S-nitrosylation of the conserved Cys^162^ residue (Figure 3), which is another type of post-translational modification of BIN2 (Wang et al., 2015). Interestingly, it was postulated that due to localization of the Cys^162^ residue in the BIN2 protein structure, its S-nitrosylation could disrupt the interaction between BIN2 and the BES1/BZR1 transcription factors (Mao and Li, 2020). Interestingly, the Cys^162^ residue is also oxidized by ROS (as described above) (Figure 3), and apparently oxidation of this residue results in the activation of BIN2, while NO-mediated S-nitrosylation of this residue inactivates BIN2. Thus, in this case an interesting mechanism of competitive post-translational modification of BIN2 with the opposite effect on its activity can be observed. The Cys^162^ residue is conserved among the GSK homologs (Supplementary Figure 1), what indicates that this regulatory mechanism was of crucial importance during the plant evolution. However, further research is required to fully elucidate this aspect.

Activity of the GSK proteins is also modulated by regulation of their subcellular localization. Initially, it was reported that recruitment of BIN2 to plasma membrane influences the BR signaling. One of the regulators of the subcellular localization of the BIN2 kinase is plasma membrane-associated OCTOPUS (OPS) protein involved in phloem development, which is expressed in provascular cells and phloem initials. The OPS protein is a positive regulator of protophloem differentiation in the root meristem (Truernit et al., 2012; Rodriguez-Villalon et al., 2014). Interestingly, OPS plays its role as negative regulator of the BIN2 activity, as well. OPS directly interacts with BIN2 which results in higher accumulation of the BIN2 kinase at the plasma membrane, and its lower accumulation in the nucleus (Figure 4). Therefore, through recruiting BIN2 to the plasma membrane OPS may suppress the inhibitory effect of BIN2 on its substrate BES1/BZR1 transcription factors in the nucleus (Anne et al., 2015). Hence, it may indicate a positive role of OPS in regulating the BR signaling to enhance phloem differentiation, and the importance of BIN2 subcellular localization for its proper activity (Li et al., 2021). However, an alternative explanation has been also suggested (Kang et al., 2017). Although the protophloem sieve element differentiation is impaired in the *bri1 brl1 brl3* triple mutant, it was proposed that this effect might not be regulated by canonical downstream BR signaling components. The effect observed in the triple mutant proved to be moderate when compared with the *ops* mutants. Thus, it was suggested that the OPS function may not proceed entirely through BIN2 and its target proteins (Kang et al., 2017).

Interestingly, it was reported that the BIN2 homolog in rice, OsGSK2, is regulated by the protein product of the *GW5* (Grain Width5, QTL for grain width and weight) gene in the same manner. GW5 is a transmembrane protein which recruits OsGSK2 to the plasma membrane what results in suppression of the negative impact of OsGSK2 on the BR signaling components in the nucleus (Figure 4). It was reported that GW5 acts in the BR signaling pathway, but also regulates important agronomical traits in rice−grain width and weight (Liu et al., 2017).

Recently, another mechanism of the GSK3 kinase regulation through altering its localization has been proposed in *Sorghum bicolor*. It was demonstrated that the Dwarfing1 (DW1) protein directly interacts with Sorghum ortholog of BIN2 – SbBIN2. In the presence of DW1 protein the level of SbBIN2 in the nucleus noticeably decreased, in contrast to the accumulation of transcription factor BZR1 which remained unchanged. Although the DW1 protein localizes to the plasma membrane and the cytosol, SbBIN2 is negatively regulated by DW1 through inhibition of nuclear localization of SbBIN2 and triggering the export of the SbBIN2 kinase from nucleus (Hirano et al., 2017; Figure 4). However, further research is needed to fully clarify the DW1 role in regulation of the SbBIN2 activity, including identification of other components of the molecular mechanism.

Another mechanism of the BIN2 activity regulation has been described in the vascular tissue of hypocotyls in Arabidopsis. In this mechanism the BIN2 activity is regulated by the Tracheary Element Differentiation Inhibitory Factor (TDIF) and its receptor TDR (TDIF-TDR module). Unlike the BR signaling, in the TDIF signaling BIN2 is directly activated by TDIF-TDR interaction to repress the xylem differentiation from procambial cells in hypocotyls. Interestingly, TDIF-TDR-BIN2 module inhibits the activity of the transcription factor BES1, which is known to enhance xylem differentiation, whereas BZR1 is not regulated in this manner. In addition, it was indicated that the cytoplasmic kinase domain of TDR is able to interact with all GSK3 kinases from the subgroups I and II, and also with AtSK32 and AtSK41. However, the interaction is noticeably weaker in the case of the last two kinases (Kondo et al., 2014).

It was reported that another plasma membrane-associated proteins, Tetratricopeptide Thioredoxin-like3 (TTL3) and Brassinosteroid Signaling Kinase3 (BSK3) interact with the BIN2 kinase at the plasma membrane in Arabidopsis. It was suggested that these interactions may stimulate the early BR signaling events. It was also suggested that the TTL3-mediated recruiting the BIN2 kinase at the plasma membrane may at least partly promote the BR signaling through enhancement of BIN2 degradation (Amorim-Silva et al., 2019; Ren et al., 2019). Activity of the OPS, TTL3 and BSK3 proteins and their inhibitory effect on BIN2 is stimulated by BR (Li et al., 2021; Figure 4).

The subcellular localization of the GSK proteins is also dynamically altered during stomatal development in Arabidopsis. Before asymmetric cell division takes place, the BIN2 and AtSK12 proteins are recruited in the vicinity of cell membrane by the scaffold membrane protein Polar Localization During Asymmetric Division and Redistribution (POLAR) and the Breaking of Asymmetry in the Stomatal Lineage (BASL) protein (Figure 4). This relocalization of GSKs allows the asymmetric cell division. After the asymmetric cell division, the GSK proteins dissociate from POLAR and are accumulated in the nucleus (Pillitteri et al., 2011; Houbaert et al., 2018). In this subcellular shuttle of the GSK proteins the BSL1 protein is involved, which is known to enhance the BR signaling (Mora-Garcia et al., 2004; Kim et al., 2009, 2018). BSL1 suppresses the interaction between BIN2 and BASL near the plasma membrane (Guo et al., 2021; Li et al., 2021).

Activity of the BIN2 kinase through subcellular localization is also modulated by the Heat shock protein 90 (HSP90). Research conducted during the last decades provided an insight into a role of this highly conserved chaperone protein and its involvement in stress response and plant development (Milioni and Hatzopoulos, 1997; Margaritopoulou et al., 2016; Li et al., 2020b). HSP90 interacts with BIN2 in the nucleus, affecting the BIN2 function (Samakovli et al., 2014). Application of the HSP90 inhibitor, geldanamycin (GDA), caused translocation of that protein from the nucleus to the cytoplasm, and the accumulation of BIN2 in the nucleus decreased as well. This intriguing result indicated that the presence of HSP90 is important for the BIN2 nuclear localization and activity (Figure 4). Samakovli et al. (2014) pointed out that under the GDA treatment, the expression level of the BR biosynthesis genes *CPD1* and *DWF4* decreased, which indirectly indicated that the BIN2 activity was not sustained by HSP90. Therefore, active HSP90 positively regulates the BIN2 function. However, analysis of the relationship between HSP90, BIN2 and BL demonstrated that there is a link between these components, since the presence of BL induces the export of HSP90-BIN2 complex to the cytoplasm. Moreover, several studies confirmed that HSP90 is able to interact with the BES1 transcription factor (Shigeta et al., 2015; Samakovli et al., 2020), what remains to be further investigated.

Subcellular shuttle of BIN2 may also constitute a switch between physiological adaptations of plants during exposure to salinity stress, as well as during recovery period. Upon exposure to the salinity stress, calcium signaling pathway is activated by calcium sensors Salt Overly Sensitive3 (SOS3) and SOS3-like Calcium Binding Protein8 (SCaBP8) which activate SOS2 protein kinase and recruit it to the plasma membrane (Halfter et al., 2000; Quan et al., 2007). As a consequence, SOS2 phosphorylates SOS1 which functions as Na^+^/H^+^ antiporter exporting the Na^+^ ions to the apoplast (Lin et al., 2009; Yang and Guo, 2018). Under the salinity stress conditions, BIN2 plays its normal role of negative regulator of the BR signaling in the nucleus. However, during the recovery period the salinity-triggered calcium signaling is suppressed, and BIN2 is recruited to the plasma membrane by SOS3 and SCaBP8 to phosphorylate and inhibit SOS2 in order to attenuate the salinity-stress response (Li et al., 2020c; Figure 4). Thus, the subcellular shuffle of BIN2 between the nucleus and the plasma membrane vicinity is important for efficient transition from physiological response to the salinity-stress to recovery of plant growth when vegetation conditions become normal again (Li et al., 2021).

## Involvement of glycogen synthase kinases in various signaling pathways regulating diverse physiological processes and plant yield

Plant GSKs function as hubs interconnecting various signaling pathways regulating developmental processes and responses to environmental stresses through interacting with various upstream regulators and phosphorylating various types of substrate proteins, including a large group of transcription factors, kinases participating in diverse signaling pathways, metabolic enzymes, scaffold and cytoskeleton proteins, cyclins, and proteins participating in the proteasomal degradation (Youn and Kim, 2015). As a result of these extensive phosphorylations, GSKs influence activity, stability, and subcellular localization of target proteins participating in various physiological processes. Phosphorylation sites of several protein substrates of GSKs and impact of these phosphorylations on the substrates’ activity, stability and localization have been recently described (Youn and Kim, 2015; Li et al., 2021). An illustration of the versatile roles of the GSK family proteins is BIN2 which participates in the interplay of various signaling pathways, mediates drought, cold, and salinity stress responses, and regulates metabolic processes. Generally, plant GSK proteins regulate various physiological and developmental processes, including cell growth, root development, stomata patterning, xylem differentiation, flowering, as well as response to light and stress factors (Youn and Kim, 2015). Recent reports indicate that they are also involved in fruit and grain development in dicot and monocot crop species, respectively. Molecular mechanisms of this regulation will be described below. Some of these functions are executed redundantly with other representatives of the GSK family. Noteworthy, genes encoding the GSK3 proteins generally show a broad expression pattern in various plant organs. However, some representatives of this family are expressed in specific organs or at specific developmental stages (Li et al., 2021). It should be also kept in mind that different members of the GSK family may have overlapping, but non-identical and sometimes specific functions (Youn et al., 2013).

### Effects of glycogen synthase kinases on activity of substrate proteins regulating various physiological and developmental processes

**FIGURE 5:**
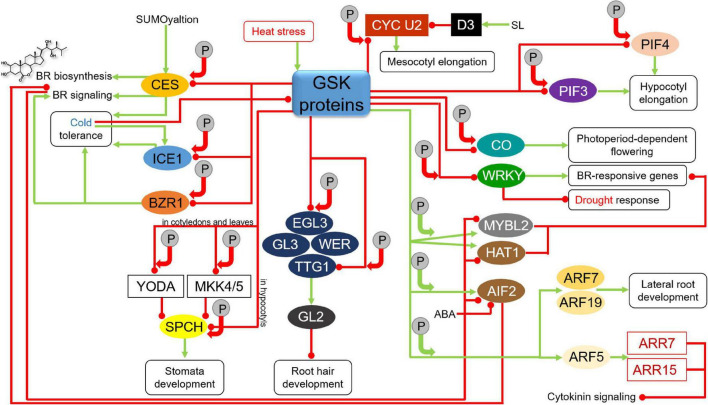
Influence of GSKs on proteins participating in various developmental, hormonal, and stress response pathways. Green arrows indicate stimulation, whereas red lines with the bullet points represent suppression. Solid circles containing the letter “P” denote phosphorylation events. Positive and negative effects of the phosphorylations on the target proteins are represented by green and red arrows, respectively. Detailed description is given in the text. CES, CESTA; ICE1, Inducer of CBF Expression1; BZR1, Brassinazole-resistant1; YODA, Mitogen-Activated Protein Kinase; MKK4/5, Mitogen-Activated Protein Kinase kinase 4/5; SPCH, SPEECHLESS; EGL3, Enhancer of Glabra3; GL3, Glabra3; WER, WEREWOLF; TTG1, Transparent Testa Glabra1; GL2, Glabra2; CYC U2, U-type cyclin; D3, Dwarf3 ubiquitin ligase; SL, strigolactone; CO, CONSTANS; WRKY, Trp-Arg-Lys-Tyr domain transcription factor; MYBL2, Myeloblastosis Family Transcription Factor-like2; HAT1, Homeodomain-leucine zipper protein Arabidopsis thaliana1; AIF2, ATBS1-Interacting Factor2; ABA, abscisic acid; PIF, PHYTOCHROME INTERACTING FACTOR; ARF, Auxin Response Factor; ARR, Arabidopsis Response Regulator.

In a number of cases the GSK3-mediated phosphorylations lead to degradation of target transcription factors regulating various developmental processes in Arabidopsis and rice. As the major component of the BR signaling pathway, BIN2 regulates stability of various proteins involved in regulation of the BR biosynthesis and signaling in the model and crop species (Guo et al., 2013; Gruszka, 2018, 2020). BIN2 phosphorylates CESTA (CES) transcription factor which functions as a positive component of the BR signaling and positively regulates the rate of BR biosynthesis (Figure 5). CESTA is mainly involved in maintaining the BR homeostasis at an early stage of plant development (Poppenberger et al., 2011; Gruszka, 2019). Noteworthy, expression of genes which encode the CPD and DWF4 enzymes, which catalyze the rate-limiting steps of the BR biosynthesis, is strongly suppressed by the major BR-regulated transcription factors BZR1 and BES1 (Wang et al., 2002; He et al., 2005). This BIN2-mediated phosphorylation of the CES transcription factor leads to a decrease in its stability and activity, and additionally represses its SUMOylation dependent nuclear localization (Khan et al., 2014). Interestingly, the BIN2-mediated phosphorylation of CES which influences its stability and compartmentalization is antagonistic to SUMOylation of adjacent amino acid residues, and this antagonistic post-translational modifications may be responsible for efficient regulation of the BR biosynthesis (Poppenberger et al., 2011). It is also known that the CES transcription factor is also involved in plant response to cold stress, therefore, the BIN2-mediated inactivation of the CES transcription factor reduces cold tolerance (Eremina et al., 2016). Additionally, the BIN2 kinase and its homologs perform phosphorylation of the Inducer of CBF Expression1 (ICE1) transcription factor which positively regulates plant response to cold stress (Liu et al., 2018; Lv and Li, 2020) and the BZR1 transcription factor, which apart from being one of the major transcriptional regulators of the BR-dependent gene expression (Wang et al., 2002; Gruszka, 2018), also positively regulates freezing tolerance (Li et al., 2017). The BIN2-mediated phosphorylation of the ICE1 transcription factor promotes its interaction with the High Expression of Osmotically Responsive Gene1 (HOS1) ubiquitin kinase (Ding et al., 2015; Ye et al., 2019). The GSK-mediated phosphorylation of these transcription factors initiates their degradation and reduced cold tolerance which enables coordination of the processes of plant growth and response to the cold stress (Eremina et al., 2016; Li et al., 2017, 2021; Ye et al., 2019). On the other hand, it was recently suggested that the cold stress stimulates the ICE1 transcription factor activity directly and indirectly by inhibition of the BIN2 kinase activity (Lv and Li, 2020; Figure 5). Although the accumulating body of evidence suggests involvement of the GSK proteins in the cold stress tolerance, further research is needed to fully elucidate mechanisms regulating the GSK activity which enable efficient coordination of the growth and cold-stress tolerance processes (Mao et al., 2021).

The GSK3-mediated phosphorylation influences activity of various proteins involved in regulation of developmental processes in the BR-dependent manner. One of these processes is root hair development. In Arabidopsis, root hair development is regulated by a group of transcription factors WEREWOLF (WER), Glabra3 (GL3), its homolog Enhancer of Glabra3 (EGL3) and Transparent Testa Glabra1 (TTG1) (Galway et al., 1994; Lee and Schiefelbein, 1999; Bernhardt et al., 2003). The WER-GL3/EGL3-TTG1 complex of transcription factors enhances expression of the *Glabra2* (*GL2*) gene and accumulation of the encoded transcription factor to suppress expression of root hair-specific genes. BIN2 phosphorylates EGL3 to suppress its nuclear localization in root hair cells, prevent formation of the WER-GL3/EGL3-TTG1 complex and inhibit the *GL2* gene expression. It was also reported that BIN2 phosphorylates the TTG1 transcription factor to repress activity of the WER-GL3/EGL3-TTG1 complex what results is stimulation of root hair development (Figure 5). In this way, the BR-dependent inactivation of BIN2 has an impact on root hair development (Cheng et al., 2014).

The GSK3-dependent phosphorylation regulates also activity of proteins involved in stomata development. In Arabidopsis BIN2 phosphorylates and deactivates Mitogen-Activated Protein Kinase (MAPK) kinases YODA and MAPK kinase 4/5 (MKK4/5) in the cell cortex. It results in suppression of the MAPK signaling to indirectly stimulate activity of the SPEECHLESS (SPCH) transcription factor in the nucleus to promote asymmetric cell division and stomata formation in cotyledons and leaves (Kim et al., 2012; Khan et al., 2013). On the other hand, BIN2 phosphorylates the SPCH transcription factor in the nucleus and promotes its degradation to enhance cell differentiation and repress stomata development in hypocotyls (Gudesblat et al., 2012; Figure 5). In order to effectively coordinate these two molecular mechanisms, subcellular localization of BIN2 needs to be precisely regulated by dynamic interactions of BIN2 with the two above-mentioned scaffold proteins POLAR and BASL in the cell cortex (Houbaert et al., 2018; Guo et al., 2021; Li et al., 2021). Interestingly, it is also known that BIN2 phosphorylates the POLAR protein to reduce its stability and the BASL protein to regulate its mobility and localization (Zhang Y. et al., 2016; Houbaert et al., 2018). Thus, the regulatory mechanism between the BIN2 kinase and the POLAR and BASL proteins is mutual.

Another protein target of the BIN2 kinase is the PHYTOCHROME INTERACTING FACTOR4 (PIF4) which controls timing of the hypocotyl elongation in a light-dependent manner (Leivar and Quail, 2011). BIN2 kinase phosphorylates and deactivates PIF4 to repress hypocotyl elongation. Interestingly, the BIN2-mediated deactivation of PIF4 occurs mainly at dawn and correlates with diurnal rhythm of hypocotyl elongation (Bernardo-García et al., 2014). Another representative of the PIF family of transcription factors, PIF3, also undergoes the BIN2-mediated phosphorylation which results in proteasome-dependent degradation of PIF3 during plant development in the darkness (Ling et al., 2017; Figure 5). It was reported that BIN2-mediated phosphorylation results in deactivation of Cellulose SynthaseA1 (CESA1). It is suggested that this deactivation is responsible for reduced cellulose synthesis which is observed in the BR mutants of Arabidopsis (Sanchez-Rodriguez et al., 2017).

Another developmental process which is regulated on the basis of GSK3-mediated phosphorylation of target proteins is floral transition. Interestingly, the aforementioned gain-of-function mutant *bin2-1* of Arabidopsis exhibits repressed floral organ elongation. On the basis of analysis of this mutant, it was reported that BIN2 negatively regulates floral development in Arabidopsis through influencing auxin accumulation (Li et al., 2020d). One of the GSK3s in Arabidopsis, AtSK12, phosphorylates the CONSTANS (CO) transcription factor which is a key regulator of flowering time related with photoperiod. During the floral transition AtSK12 phosphorylates the CO transcription factor what results in degradation of CO in leaf vascular tissue, and this mechanism represses the photoperiod-dependent flowering (Chen et al., 2020; Figure 5). Noteworthy, BIN2 and its homologs indirectly influence flowering time through their negative impact on the activity of the BZR1 and BES1 transcription factors which are known to modulate expression of several key flowering regulators (Li and He, 2020). A very recent report indicates another interconnection between the GSK proteins and flowering regulation. Interestingly, both gain-of-function *bin2-1* mutation and overexpression of the *BIN2* gene resulted in early flowering, whereas the BIN2 loss-of-function mutant *bin2-3 bil1 bil2* showed late-flowering phenotype. On the other hand, BIN2 plays a major role in mediating the crosstalk between the BR signaling and response to heat stress. It was reported that heat shock increased the cellular abundance of the BIN2 protein (Figure 5). Thus, it was suggested that under the heat shock conditions the BIN2 activity is enhanced to promote early flowering and allow plant reproduction. However, it occurs at the expense of thermotolerance and survivability of plants. Noteworthy, it was also proposed that BIN2 likely represses thermotolerance and enhances flowering via different target proteins (Ren et al., 2022).

The BIN2-mediated phosphorylation which results in destabilization of target proteins is also involved in mechanism regulating balance between plant growth and response to another environmental stress – drought. In this case the target proteins belong to the WRKY (Trp-Arg-Lys-Tyr domain) family of transcription factors. The WRKY transcription factors are negative regulators of drought response. Under normal growth conditions, these transcription factors cooperate with BES1 to enhance expression of the BR-responsive genes (related with plant growth), but to suppress expression of the drought-responsive genes. However, under conditions of water shortage the WRKY factors are phosphorylated and destabilized by the BIN2 kinase. In this way, under the stress conditions the BR-stimulated plant growth is repressed, but expression of drought-responsive genes is induced (Chen et al., 2017; Figure 5).

It was reported in rice that OsGSK2 regulates length of mesocotyl through phosphorylation of the CYC U2 cyclin. Brassinosteroids promote the mesocotyl elongation in rice by repressing the OsGSK2-mediated phosphorylation of the U-type cyclin CYC U2 which controls cell division. The OsGSK2-mediated phosphorylation of CYC U2 reduces its stability, and consequently suppresses cell division in rice mesocotyl. However, mesocotyl elongation in rice is negatively regulated by another phytohormone, strigolactone (SL), through activity of the F-box Dwarf3 (D3) ubiquitin ligase which functions as the major positive regulator of the SL signaling. The D3 ubiquitin ligase interacts with the phosphorylated CYC U2 cyclin to initiate its degradation. Therefore, in this case OsGSK2 integrates the BR and SL signaling pathways and allows coordinated regulation of mesocotyl length in rice by these two phytohormones (Figure 5). Moreover, it was suggested that this mechanism was also important in the process of rice domestication. Natural alleles of the *OsGSK2* gene were selected to regulate the rice mesocotyl length by coordinating the BR and SL signaling pathways (Sun et al., 2018; Gruszka, 2020).

However, it is also known that the GSK3-mediated phosphorylation may have a stabilizing effect on target proteins. Moreover, the list of target proteins in Arabidopsis which are stabilized as a result of the BIN2-mediated phosphorylation has extended over the recent years (Li et al., 2021). Examples of this group of target proteins are two transcription factors – Myeloblastosis Family Transcription Factor-like2 (MYBL2) and Homeodomain-leucine zipper protein Arabidopsis thaliana1 (HAT1). Both these transcription factors act as negative regulators of the BR-repressed gene expression (Ye et al., 2012; Zhang D. et al., 2014). It was reported that expression of the *MYBL2* gene is attenuated by BR in the BES1-dependent manner. Interestingly, MYBL2 interacts with BES1 to down-regulate the BR-repressed gene expression. Importantly, suppression of the BR-repressed gene expression is crucial for optimal BR response. Thus, MYBL2 functions as a positive regulator of the BR-dependent plant growth and acts as a components of a feedback mechanism. The BIN2-mediated phosphorylation stabilizes MYBL2 (Ye et al., 2012). It was reported that HAT1 exists mostly in its phosphorylated form under normal growth conditions. Expression of the *HAT1* gene is decreased in response to BR. It is known that HAT1 interacts with BES1 and these transcription factors bind to promoter sequences of some BR-repressed genes to synergistically suppress expression of the BR-repressed genes. Thus, it may be concluded that HAT1, similar to MYBL2, is positively involved in the BR responses. Moreover, under specific conditions BIN2 may function as a positive regulator of the BR response through its stabilizing influence on the MYBL2 and HAT1 transcription factors (Zhang D. et al., 2014; Figure 5).

The BIN2-mediated phosphorylation stabilizes also another transcription factor−ATBS1-Interacting Factor2 (AIF2). However, in this case the protein substrate of BIN2 is a negative regulator of the BR signaling. The AIF2 expression is significantly suppressed by the BR signaling in the BZR1-dependent manner. Moreover, AIF2 is also regulated at the post-translational level – BR promotes dephosphorylation of AIF2 and its subsequent degradation. Interestingly, the AIF2 degradation is enhanced by both BR and ABA. Thus, the BIN2-mediated phosphorylation stabilizes the AIF2 transcription factor and promotes its negative impact on the BR signaling (Kim et al., 2017; Figure 5).

The BIN2 protein is also involved in lateral root development, and in this case, phosphorylation catalyzed by this kinase leads to activation of target proteins. It is also an example of the role of BIN2 kinase as the hub interconnecting signaling pathways of various phytohormones. In this process the BIN2-mediated phosphorylation activates Auxin Response Factors ARF7 and ARF19 by inhibiting their interaction with Auxin/Indole-3-acetic acid (Aux/IAA) transcription repressors (Cho et al., 2014). As a consequence, it activates the Lateral organ Boundaries-Domain16/Asymmetric Leaves-like18 (LBD16/ASL18) and LBD29/ASL16 proteins which enhance lateral root development (Okushima et al., 2007; Figure 5).

Another member of the GSK3 family, AtSK23/BIL1, regulates xylem differentiation and cambial cell identity. Interestingly, the AtSK23/BIL1 regulates this process through modulating activity of the Auxin Response Factor5 (ARF5). It was reported that AtSK23/BIL1 phosphorylates ARF5 and enhances its inhibitory influence on the vascular cambial activity through activation of two Arabidopsis Response Regulators (ARRs), ARR7 and ARR15, which are negative regulators of cytokinin signaling (Han et al., 2018; Mao et al., 2021). This is another illustration of the involvement of the GSK3 proteins in the multi-level interhormonal crosstalk which regulates the developmental process (Figure 5).

**FIGURE 6:**
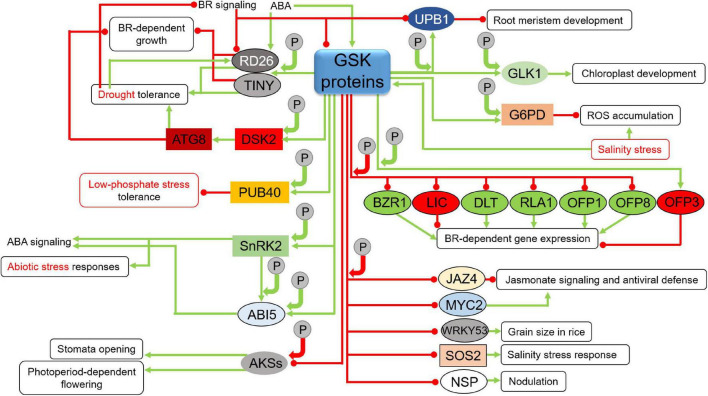
Influence of GSKs on proteins participating in various developmental, hormonal, and stress response pathways (continuation). Green arrows indicate stimulation, whereas red lines with the bullet points represent suppression. Solid circles containing the letter “P” denote phosphorylation events. Positive and negative effects of the phosphorylations on the target proteins are represented by green and red arrows, respectively. Detailed description is given in the text. ATG8, Autophagy8; RD26, Responsive to Desiccation26; TINY, transcription factor; DSK2, Dominant Suppressor of KAR2; PUB40, Plant U-Box40 ubiquitin ligase; SnRK2, Sucrose non-fermenting1-Related protein Kinase2; ABI5, Abscisic acid Insensitive5; AKSs, ABA-responsive Kinase Substrates; UPB1, UPBEAT1; GLK1, GOLDEN2-like1; G6PD, Glucose-6-phosphate dehydrogenase; BZR1, Brassinazole-resistant1; LIC, Leaf and Tiller Angle Increased Controller; DLT, DWARF and Low-Tillering; RLA1, Reduced Leaf Angle1; OFP, Ovate Family Protein; JAZ4, Jasmonate ZIM-Domain4; MYC2, Myelocytomatosis2 transcription factor; WRKY53, Trp-Arg-Lys-Tyr domain transcription factor53; SOS2, Salt Overly Sensitive protein kinase2; NSP, Nodulation Signaling Pathway transcription factor.

Another proteins which are stabilized through the BIN2-mediated phosphorylation are the TINY and Responsive to Desiccation26 (RD26) transcription factors which participate in a crosstalk between the BR signaling and stress response (Jiang et al., 2019; Xie et al., 2019). In this signaling crosstalk, the TINY transcription factor represses the BR-dependent growth through antagonistic interaction with the BES1 transcription factor. However, TINY positively regulates the drought tolerance by enhancing expression of drought-responsive genes. Under drought conditions BIN2 phosphorylates TINY which activates many drought-responsive genes (Sun et al., 2008; Xie et al., 2019). The BIN2-mediated phosphorylation stabilizes the TINY transcription factor and promotes its role in drought tolerance (Xie et al., 2019). As far as the RD26 transcription factor is concerned, its expression is suppressed by the BR signaling, however, its activity is enhanced by drought and ABA. BIN2 positively regulates drought response by phosphorylating and stabilizing RD26 which interacts antagonistically with the BES1 transcription factor to repress expression of the BES1 target genes, which are related with plant growth, but to promote expression of drought-responsive genes. The BIN2-mediated phosphorylation and stabilization of RD26 is induced by ABA. Under drought conditions the BR signaling is attenuated what results in the enhancement of the *RD26* gene expression and accumulation of the encoded protein. The accumulating RD26 binds the BES1 transcription factor to further suppress the BR-dependent gene expression (Chung et al., 2014; Ye et al., 2017; Jiang et al., 2019; Figure 6). Another illustration of the involvement of the GSK3 proteins in the interplay between the BR and stress responses is the BIN2-mediated phosphorylation and activation of the Dominant Suppressor of KAR2 (DSK2) which functions as ubiquitin receptor protein. The BIN2-mediated phosphorylation of DSK2 facilitates its interaction with the Autophagy8 (ATG8) protein which is a prerequisite for degradation of the BES1 transcription factor – major regulator of the BR-dependent gene expression. This positive influence of the BIN2 kinase on the DSK2-ATG8 module improves plant tolerance to drought and nutrient-deficiency stress (Nolan et al., 2017). BIN2 performs phosphorylation-dependent stabilization of another component of the ubiquitin pathway – the Plant U-Box40 (PUB40) ubiquitin ligase. The phosphorylated and stabilized PUB40 ubiquitin ligase interacts with the BZR1 transcription factor to mediate its degradation specifically in roots. However, in this case the PUB40-mediated degradation of BZR1 compromises tolerance of Arabidopsis roots to low-phosphate stress (BZR1 accumulation in the triple mutant *pub39 pub40 pub41* roots leads to resistance to low phosphate availability) (Kim et al., 2019). Interestingly, it seems that BIN2 may alternatively influence plant responses to the environmental stresses−improving plant tolerance to drought and nutrient-deficiency stress through activation of the DSK2-ATG8 module or compromising tolerance of roots to the low-phosphate stress via stabilization of the PUB40 ubiquitin ligase (Figure 6). However, explanation of this differential regulation of stress responses may be root-specific activity of PUB40. In rice, OsGSK2 stabilizes ubiquitin ligase OsPUB24 in the phosphorylation-dependent manner to promote degradation of OsBZR1 in proteasome (Min et al., 2019). However, it is not yet known whether it influences response of rice plants to environmental stresses.

It is known that in Arabidopsis BIN2 positively regulates ABA responses during seed germination and plant growth, and that ABA activates the BIN2 kinase (Hu and Yu, 2014). Interestingly, the gain-of-function Arabidopsis mutant *bin2-1* is hypersensitive to ABA and showed the ABA-induced gene expression pattern (Li et al., 2001; Cai et al., 2014). This indicates that BIN2 is a positive mediator of the ABA signaling (Hu and Yu, 2014; Youn and Kim, 2015). It is also known that the BIN2-mediated phosphorylation activates the Sucrose non-fermenting1-Related protein Kinase2.3 (SnRK2.3) which is a positive regulator of the ABA signaling and abiotic stress responses. The activated SnRK2.3 phosphorylates its downstream substrate ABA Response Elements-Binding Factor2 (ABF2). In this way, BIN2 positively regulates the ABA signaling (Cai et al., 2014). BIN2 phosphorylates and stabilizes also the Abscisic acid Insensitive5 (ABI5) transcription factor which is an another component of the ABA signaling that regulates seed germination. Interestingly, BIN2 and the above-mentioned SnRK proteins phosphorylate ABI5 at different amino-acid positions, and the BIN2-mediated phosphorylation and stabilization of ABI5 is dependent on the SnRK-mediated phosphorylation of ABI5. The BIN2-mediated stabilization of ABI5 positively regulates the ABA response during seed germination, whereas the BR signaling counteracts this mechanism (Hu and Yu, 2014; Figure 6). The crosstalk between BR and ABA is also illustrated by role of the BIN2 protein in regulation of three ABA-responsive Kinase Substrates (AKSs) transcription factors in Arabidopsis (Bu et al., 2017). It is known that the AKSs proteins in their unphosphorylated form stimulate stomata opening through enhancing transcription of genes which encode potassium channels (Takahashi et al., 2013). Interestingly, two of the AKS proteins (in their unphosphorylated form) regulate flowering in the photoperiod-dependent manner as well (Ito et al., 2012). It was reported that ABA promotes phosphorylation of these AKS transcription factors which is mediated by the BIN2 protein. In this case, the ABA stimulation of the BIN2 activity is mediated by the SnRK2 kinase. In this crosstalk mechanism BIN2 is involved in ABA-dependent repression of stomata opening and flowering, and this BIN2 activity is suppressed by BR (Bu et al., 2017; Figure 6).

The BIN2-mediated phosphorylation stabilizes also another transcription factors which regulate important developmental processes – UPBEAT1 (UPB1) which is involved in root development through regulation of balance between cell division and differentiation in root meristem (Li et al., 2020e) and GOLDEN2-like1 (GLK1) which participates in chloroplast development during de-etiolation (Zhang D. et al., 2021). As far as regulation of the UPB1 function is concerned, it was reported that expression of the *UPB1* gene is suppressed by BR at the transcriptional level in the BES1-dependent manner. Interestingly, the BIN2-mediated phosphorylation and activation of UPB1 causes impairment of the root meristem development. Thus, it was postulated that BRs promote root growth through suppression of activity of the BIN2-UPB1 module (Li et al., 2020e). It was reported that BIN2 phosphorylates GLK1 which results in enhancement of its stability and activity, and as a consequence stimulates chloroplast development (Waters et al., 2008; Zhang D. et al., 2021; Figure 6). One of the GSK3-like proteins in Arabidopsis, AtSK11, phosphorylates the Glucose-6-phosphate dehydrogenase (G6PD). This phosphorylation stimulates the G6PD activity and represses the reactive oxygen species (ROS) accumulation which is induced by the salinity stress. Interestingly, the salinity stress induces the AtSK11 kinase activity, and overexpression of the *AtSK11* gene leads to improved tolerance to salinity stress and response to pathogen attack (Dal Santo et al., 2012; Stampfl et al., 2016; Li et al., 2021). It is also known that expression of the *AtSK22/BIL2* gene in Arabidopsis is specifically induced by NaCl and ABA, and overexpression of this gene results in a constitutive activation of a group of salinity responsive genes and improves the stress tolerance (Piao et al., 2001). Moreover, it was reported that the salt stress induces expression of three other GSK3 genes – *AtSK13*, *AtSK31* and *AtSK42* (Charrier et al., 2002). Moreover, induction of expression of the *GSK* genes by salt stress was observed also in crops species, such as wheat, rice and sugarcane (*Saccharum officinarum*) (Chen et al., 2003; Koh et al., 2007; Patade et al., 2011; Figure 6).

In rice, the OsSK/GSK3 proteins (mostly OsGSK2) are major negative regulators of several transcription factors, such as BZR1, Leaf and Tiller Angle Increased Controller (LIC), DWARF and Low-Tillering (DLT), Reduced Leaf Angle1 (RLA1), as well as Ovate Family Proteins 1 and 8 (OFP1 and 8) which are all directly involved in the BR-dependent gene expression. The BZR1, DLT, RLA1, OFP1 and 8 transcription factors are positive regulators of the BR-dependent gene expression, whereas LIC is a negative regulator of this process (Tong et al., 2012; Zhang C. et al., 2012; Yang et al., 2016; Qiao et al., 2017; Xiao et al., 2017; Min et al., 2019; Gruszka, 2020; Figure 6). Interestingly, the BZR1 and LIC transcription factors mutually regulate their expression in an adverse manner, and this mechanism is dependent on the BR concentration. The BZR expression is induced at low BR concentration, whereas the LIC expression is stimulated by high concentrations of BR. Importantly, this mechanism of dynamic transcriptional regulation is additionally modulated by activity of the OsSK/GSK3 proteins (Zhang C. et al., 2012). Recently, it has been also reported in rice that OsGSK2-mediated phosphorylation leads to degradation of the Jasmonate ZIM-Domain4 (OsJAZ4) and OsWRKY53 transcription factors which allows for interconnection of the BR signaling with jasmonic acid and mitogen-activated protein kinase signaling pathways, respectively (He et al., 2020; Tian et al., 2021). The OsGSK2-mediated phosphorylation of OsJAZ4 facilitates degradation of this transcription factor in proteasome. It should be noted that OsJAZ4 is a negative regulator of the jasmonate signaling and antiviral defense. Thus, OsGSK2 stimulates plant antiviral defense by deactivating OsJAZ4, and consequently, promoting the jasmonate signaling (He et al., 2020). Noteworthy, the OsGSK2-OsWRKY53 interaction influences grain size in rice (Tian et al., 2021; Figure 6). This diverse group of transcription factors being target proteins of the OsGSK2-mediated phosphorylation allows OsGSK2 to indirectly participate in various molecular processes regulating plant growth, development and response to environmental stresses (Li et al., 2021).

Similar to Arabidopsis, recent reports indicated that in rice the GSK3-mediated phosphorylation may also have stabilizing effect on target proteins. As far as the BR signaling is concerned, OsGSK2 phosphorylates and stabilizes another representative of the above-mentioned Ovate Family Proteins – OFP3 (Xiao et al., 2020). Interestingly, in contrast to the OFP1 and OFP8 proteins which are positive regulators of the BR-dependent gene expression (Gruszka, 2020), OFP3 is a repressor of transcription and the OsGSK2-mediated phosphorylation stabilizes this repressor at low BR concentration to repress the BR response (Xiao et al., 2020). Thus, it may be concluded that since OsGSK2 suppresses the OFP1 and OFP8 transcription factors (positive regulators of the BR-dependent gene expression), but stabilizes the OFP3 (repressor of transcription) at low BR concentration, the OsGSK2 function may allow for efficient adjustment of the BR signaling rate depending on the BR concentration (Figure 6).

### Involvement of the glycogen synthase kinase proteins in regulation of plant reactions to environmental factors

Efficient adjustment of plant developmental, physiological and metabolic processes to constantly changing environmental conditions is crucial for plant survival and reproduction. Importantly, involvement of the GSK3-like proteins in plant reaction to abiotic stresses has been reported in several model and crop species. As it was mentioned above, in Arabidopsis, the AtSK11 and AtSK22/BIL2 kinases play a positive role in the stress tolerance (Piao et al., 2001; Dal Santo et al., 2012). However, as far as reaction to the salinity stress is concerned, BIN2, BIL1 and BIL2 are negative regulators of the salinity stress response, with BIN2 playing the major role. BIN2 plays its role by phosphorylating the above-mentioned SOS2 kinase on the Thr^172^ position what results in the inhibition of the SOS2 kinase (Figure 6). The interaction between BIN2 and SOS2 is promoted by the aforementioned proteins SOS3 and SCaBP8, however, BIN2 is able to repress the SOS2 activity both in the cytoplasm and at the plasma membrane. Moreover, it is known that BIN2 functions as a negative regulator of root growth under the salinity stress conditions by phosphorylating and suppressing the SOS2 kinase. However, during the recovery phase of development, upon exposure to the salinity stress, BIN2 is recruited to the plasma membrane to deactivate the SOS2 kinase and repress the salinity-stress response. Interestingly, in this mechanism the SOS2 activity is regulated by BIN2 under the salinity-stress conditions, but also during the recovery phase. According to a recently proposed model, under normal conditions the BR signaling stimulates plant growth by restricting the BIN2 kinase localization to the plasma membrane, while activity of the BZR1 and BES1 transcription factors in the nucleus is enhanced. Concomitantly, activity of the SOS2 kinase in the cytoplasm is repressed (most likely by proteins from the 14-3-3 family). However, under the salinity stress conditions, the SOS2 kinase is recruited to the plasma membrane, where it activates the above-mentioned Na^+^/H^+^ antiporter SOS1 which exports the Na^+^ ions to the apoplast, whereas BIN2 translocates from vicinity of the plasma membrane to the nucleus, where it suppresses activity of the BZR1 and BES1 transcription factors, and consequently, the BR-dependent plant growth. At the same time, the BIN2-mediated inhibition of SOS2 prevents this protein from potential stress-induced over-activation. During the recovery phase, BIN2 is recruited to the plasma membrane again, where it suppresses the SOS2 activity and the salinity stress response, whereas activities of the BZR1 and BES1 transcription factors in the nucleus are derepressed, what results in stimulation (recovery) of plant growth. Thus, according to the recent model the subcellular shuttling of BIN2 maintains plant homeostasis through the involvement in molecular mechanism which fine-tunes the salinity stress response, and this mechanism is mediated by the crosstalk between the SOS pathway and the BR signaling-regulated plant growth (Li et al., 2020c). However, the OsGSK5/OsSK41 kinase of rice and its homolog in alfalfa, *Medicago sativa* protein Kinase4 (MsK4), were shown to promote the salinity stress tolerance in transgenic plants (Kempa et al., 2007; Thitisaksakul et al., 2017). Interestingly, in both cases this stress-tolerance effect was related with carbohydrate metabolism, an in the case of OsGSK5 it was achieved, at least partially, through preferential carbon allocation to root starch (Thitisaksakul et al., 2017). In soybean (*Glycine max*) the GSK3-like protein GmGSK, which is highly homologous to AtSK11, is induced by abiotic stresses and improves tolerance to the salinity stress (Zhang C. et al., 2010). It was also reported that two GSK3-like kinases from wheat, TaGSK1 and TaSK5, improve tolerance to salinity stress when expressed in Arabidopsis plants. In the case of TaSK5, the overexpression resulted also in an enhanced drought tolerance (He et al., 2012; Christov et al., 2014). However, some GSK3-like proteins may negatively regulate the salt (and other abiotic) stress tolerance. It was reported that knockout of the rice gene *OsGSK1/OsSK21* caused increased tolerance to several abiotic stresses, including the salinity, drought, and thermal stress. It indicated that the encoded kinase OsGSK1/OsSK21 plays a role of negative regulator of rice response to these abiotic stresses (Koh et al., 2007). In line with this report, silencing of several genes encoding the GSK proteins in barley promoted growth of barley plants under the salt stress conditions (Kloc et al., 2020). These diverse effects of the various GSK3-like proteins on plant tolerance to abiotic stresses may results from subfunctionalization among the members of the GSK family. However, it should also be kept in mind that the stress response is a complex phenomenon regulated by a number of genes and highly dependent on complicated physiological and metabolic adjustments, and therefore the GSK-dependent regulation of the stress responses may also be species-specific. Some difference between the reports may also stem from the fact that in some of the studies the GSK-like genes from one species were transgenically (over)expressed in other species.

Apart from the involvement in plant reaction to the abiotic stresses, the GSK3 proteins of various model and crop species were implicated in regulation of plant reaction to pathogen attack (Mao et al., 2021). However, the overall picture which emerges from these studies is not totally explicit. In Arabidopsis, the AtSK11 kinase is rapidly accumulated in response to exposure to pathogen, and loss of function of this kinase increased susceptibility to *Pseudomonas syringae*, what suggests a positive role of this kinase in plant immunity against this pathogen (Stampfl et al., 2016). On the contrary, in pepper (*Capsicum annum*), a putative GSK3-like kinase CaSK23 plays a negative role in regulation of plant immunity against the soil-borne bacterial pathogen *Ralstonia solanacearum.* Silencing of the *CaSK23* gene resulted in enhanced expression of immunity-associated genes and improved tolerance to this pathogen (Qiu et al., 2018). In line with this report, the alfalfa GSK-like protein MsK1 also negatively regulates plant immunity against pathogens. Interestingly, accumulation of the MsK1 protein and its activity were significantly decreased upon exposure to pathogen, and its transgenic over-expression in Arabidopsis resulted in reduced tolerance of plants to *Pseudomonas syringae* (Wrzaczek et al., 2007). Apart from the regulation of plant reaction to pathogen infection, the GSK3-like proteins are also involved in a very important process of legume plant biology – plant-rhizobium symbiosis. In *Lotus japonicus*, accumulation of two GSK3-like proteins, LSK1 and LSK2, is induced by a contact of plants with their natural symbiotic bacterium. It was reported that loss of function of the *LSK1* gene caused an increase in nodule formation. It points to a negative role of LSK1 in regulation of symbiotic nodulation (Garagounis et al., 2019). In soybean role of the GSK3-like proteins in regulation of the nodulation process is related with plant reaction to salinity. Interestingly, in this crop species accumulation of the GSK3-like proteins belonging to subgroup II is stimulated by high salinity conditions. However, these kinases repress nodulation under the salt-stress conditions. This effect results from the GSK3-mediated phosphorylation of two highly similar Nodulation Signaling Pathway (NSP) transcription factors which reduces their DNA-binding capability, and as a consequence, precludes symbiotic nodulation under the salt-stress conditions (He et al., 2021; Mao et al., 2021; Figure 6).

The GSK3-like kinases are also involved in regulation of plant reactions to viral infections (Mao et al., 2021). It was recently reported in rice that the OsGSK2/OsSK22 kinase phosphorylates the Jasmonate ZIM-domain4 (OsJAZ4) transcription factor to prevent its association with another transcription regulator, OsJAZ11, and formation of the OsJAZ4-OsNINJA corepressor complex. It results in degradation of OsJAZ4 that negatively regulates the jasmonate signaling and immunity response (He et al., 2020). Interestingly, the OsGSK2/OsSK22 kinase of rice phosphorylates also the Myelocytomatosis2 (OsMYC2) transcription factor which is a positive regulator of the jasmonate signaling pathway (Uji et al., 2016). The OsGSK2/OsSK22-mediated phosphorylation leads to the OsMYC2 degradation and decline in the JA-dependent tolerance to rice stripe virus (Hu et al., 2020). Thus, it seems that the OsGSK2/OsSK22-mediated phosphorylation and repression may affect both negative and positive regulators of the jasmonate signaling. This double regulation mechanism may allow for fine-tuning of plant response to viral infection (Figure 6). In other species the GSK3-like kinases are also involved in regulation of plant reactions to viral infections, including infections caused by geminiviruses which cause significant reduction in crop production and yield (Mao et al., 2021). Interestingly, it was reported that some viral proteins are able to interact with the GSK3-like proteins of various model and crop species to initiate viral infection symptoms (Piroux et al., 2007; Mills-Lujan et al., 2015; Bi et al., 2017; Mei et al., 2018).

### Role of the glycogen synthase kinase proteins in regulation of reproductive development

**FIGURE 7:**
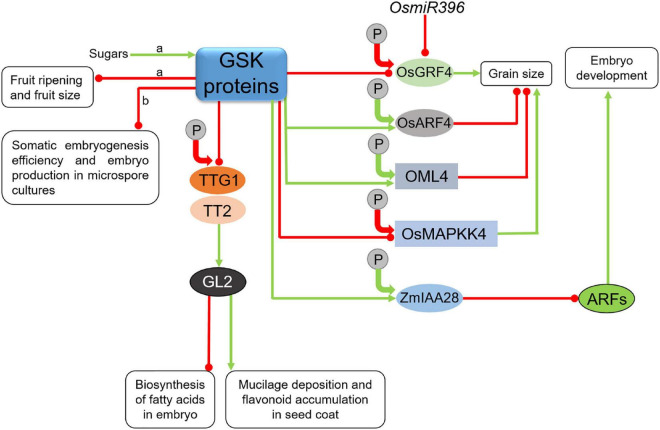
Influence of GSKs on proteins participating in various developmental and reproduction-related processes (continuation). Green arrows indicate stimulation, whereas red lines with the bullet points represent suppression. Solid circles containing the letter “P” denote phosphorylation events. Positive and negative effects of the phosphorylations on the target proteins are represented by green and red arrows, respectively. Some of the regulatory mechanisms were reported in: a, grapevine; b, rapeseed, barley, and cork oak. Detailed description is given in the text. TTG1, Transparent Testa Glabra1; TT2, Transparent Testa2; GL2, Glabra2; OsmiR396, *Oryza sativa* microRNA 396; OsGRF4, Growth Regulating Factor4; ARF, Auxin Response Factor; OML4, MEI2-like Protein4; OsMAPKK4, Mitogen-Activated Protein Kinase Kinase4; ZmIAA28, auxin response regulator28.

Some activities of the GSK3 proteins are related with regulation of reproductive development. In Arabidopsis some of the GSK proteins, such as AtSK11, AtSK12, and AtSK41 are expressed predominantly in floral tissues (Jonak et al., 1995; Dornelas et al., 2000). In rice, OsGSK2 phosphorylates Growth Regulating Factor4 (OsGRF4) and suppresses activity of this transcription factor which is involved in grain size control (Sun et al., 2016). The OsGSK2-mediated phosphorylation represses transcriptional activity of OsGRF4 and results in decrease in grain size and yield (Che et al., 2015). Interestingly, it was reported that OsGRF4 is regulated by OsmiR396, and mutation of the *OsGRF4* gene, which prevents the OsmiR396-directed regulation, results in moderately increased expression level of *OsGRF4* and upregulation of a large number of BR-induced genes to promote grain development. Consequently, it leads to large and heavy grains and increased grain yield (Figure 7). Importantly, these studies provided evidence for potential feasibility of modulating specific BR responses to improve plant productivity (Duan et al., 2015; Li et al., 2016). Two GSK3-like kinases of rice (OsGSK2 and OsGSK5) phosphorylate and stabilize Auxin Response Factor4 (OsARF4) and MEI2-like Protein4 (OML4) to regulate grain size and weight (Hu et al., 2018; Lyu et al., 2020). A loss-of-function mutation of the *OsARF4* gene results in larger rice grains. Suppression of the *OsGSK5* activity also enhances grain size and weight. It was reported that OsARF4 and OsGSK5 repress expression of a common set of genes, including some auxin-responsive genes, during grain development to negatively regulate grain size. Interestingly, recently identified loss-of-function mutation of *OsGSK5* (with enhancing effect on grain size) represents a rare allele that has not been extensively utilized in rice breeding before (Hu et al., 2018; Ying et al., 2018). Similar to OsARF4, a loss-of-function mutation of the *LARGE1* gene, which encodes the OML4 protein, also leads to large and heavy grains (Figure 7). It was reported that OML4 regulates grain size by restricting cell expansion in the spikelet hull (Lyu et al., 2020). Thus, it was suggested that OsARF4, OsGSK5, and OML4 may constitute promising targets for genetic improvement of grain yield in rice, and potentially other cereals. Moreover, in rice the OsGSK2 kinase phosphorylates and destabilizes the MAPK Kinase4 (OsMAPKK4) to regulate seed size and leaf erectness (Li et al., 2021; Tian et al., 2021). Both these phenotypic traits are very important for cereal breeding and yield improvement (Gruszka, 2020). Importantly, recent reports indicated that the GSK3 homolog proteins in barley and wheat also regulate grain size in these crops (Cheng et al., 2020; Kloc et al., 2020). All the above reports indicated that the GSK3 proteins in rice may modulate grain size by the phosphorylation-mediated stimulation or repression of substrate proteins participating in various signaling pathways (Figure 7).

A recent report in maize indicated that ZmSK2, a homolog of BIN2, is involved in embryonic development. A knockout mutation of this gene (obtained by the CRISPR/Cas9 system) resulted in kernels and embryos which were morphologically normal, but larger than those of the wild type, indicating that the loss of function of ZmSK2 promotes seed development. On the other hand, overexpression of the *ZmSK2* gene arrests embryonic development, however, it has little impact on the endosperm development. It was reported that ZmSK2 can phosphorylate and stabilize the ZmIAA28 transcription factor which is a primary auxin response regulator. Thus, it was postulated that the ZmSK2-mediated increase in the ZmIAA28 accumulation may inhibit the activity of ARFs, impairing the auxin signaling output. Interestingly, ZmSK2 and ZmIAA28 are highly expressed in embryos, but minimally expressed in endosperm. It may explain why the development of embryos is affected more than that of endosperm. However, it needs to be elucidated whether function of ZmSK2 in maize embryonic development is fully dependent on ZmIAA28. Therefore, it may be postulated that ZmSK2 constitutes a point of crosstalk between the BR and auxin signaling pathways which regulates seed development in maize (Wang et al., 2022; Figure 7).

The GSK3 activity may influence plant reproduction and yield through other mechanism as well. In Arabidopsis, two GSK3-like kinases, AtSK11 and AtSK12, phosphorylate the Transparent Testa Glabra1 (TTG1) transcription factor to prevent its interaction with other transcription factor Transparent Testa2 (TT2). As a consequence, TTG1 is prevented from binding to promoter of the *Glabra2* gene what results in downregulation of the *Glabra2* gene. This mechanism leads to enhanced biosynthesis of fatty acids in the embryo, but diminished mucilage deposition and accumulation of flavonoids in the seed coat. Therefore, this mechanism regulates carbon partitioning between various parts of developing seed (Li et al., 2018; Figure 7). Interestingly, it seems that the GSK-mediated regulation of the TTG1/GL2 module may regulate such diverse developmental processes as root hair development (Figure 5) as well as the embryo and seed coat development (Figure 7).

The proteins of the GSK3 family may also be involved in the process of fruit ripening (Mao et al., 2021). It was reported that activity of the GSK3-like homolog in grapevine (*Vitis vinifera*), VvSK1, is induced by sugars and participates in transport and accumulation of sugars in grapes during ripening. The sugar accumulation process is regulated by VvSK1 through modulation of activity of sugar-transporting proteins (Lecourieux et al., 2010). In a more recent study, overexpression of another grapevine homolog of GSK3, VvSK7, in tomato resulted in a delay in the fruit ripening process. On the other hand, treatment of grapevine plants with exogenous BR or inhibitor of the GSK3 kinases caused an enhancement of fruit ripening and enlargement of fruit size (Zeng et al., 2020). These results indicate that also in non-cereal crops the genes encoding the GSK3 proteins may be regarded as target for genome editing which may result in development of high-yield cultivars with improved fruit quality (Figure 7). Moreover, taking into account importance of semi-dwarf varieties for cereal crop breeding (Dockter et al., 2014; Dockter and Hansson, 2015; Gruszka, 2020), the *GSK* gene homologs in cereal crops may be promising targets of genetic manipulation to produce semi-dwarf varieties which may become a powerful tools in future breeding programs, particularly in the face of global climate changes (Saidi et al., 2012; Gruszka et al., 2020b; Liu et al., 2021).

Recently, another interlink between the function of GSK proteins and plant reproduction has been described. It was reported that application of synthetic small-molecule inhibitors of the GSK proteins increased initiation efficiency of *in vitro* somatic embryogenesis and embryo production in isolated microspore cultures of two crop species, rapeseed (*Brassica napus*) and barley (*Hordeum vulgare*), as well as in somatic embryogenesis cultures of a forest tree, cork oak (*Quercus suber*) (Figure 7). It is known that the *in vitro* regeneration systems, such as somatic embryogenesis, are essential in plant breeding, as they allow for propagation of elite genotypes, production of doubled-haploids, and plant regeneration after the gene editing or transformation experiments. However, in many crop and forest species the somatic embryogenesis methods are still inefficient. Thus, this report provided an important and promising tool for improvement of plant cell reprogramming and embryo production yield, also when applied in other crop species. It also indicated that targeting the GSK-mediated molecular pathways may become a potential strategy for improvement of crop plant regeneration and its application in future breeding programs (Berenguer et al., 2021).

## Conclusion

This comprehensive overview described the regulatory mechanisms which modulate activity of the plant GSK proteins, involvement of the GSK proteins in the numerous developmental, physiological, and stress-response pathways, and their participation in molecular processes which influence plant reproduction and yield. This emerging view indicates that the GSK proteins, which had been initially characterized as the major negative regulators of the BR signaling, became hubs interconnecting various molecular processes, and therefore, allowing for efficient adjustment of plant metabolism and physiology to constantly changing environmental conditions. Although significant knowledge about these mechanisms has been gathered, there are still many intriguing questions which await to be answered, particularly in terms of upstream and downstream signaling components which interact with the GSK proteins to influence their activity or be regulated by these kinases, as well as mechanisms of post-translational modification of GSKs and their mutual interdependence. Apart from the intriguing scientific questions, it also became apparent that genes encoding the GSK3 proteins may be regarded as target for genome editing which may result in development of high-yield cultivars with improved seed and fruit quality and increased yield in future breeding programs of cereal and non-cereal crops. This novel tool of plant breeding may be of particular importance in the face of global climate changes.

## Author contributions

DG conceived the subject and scope of the manuscript. Both authors prepared text of the manuscript, all figures and supplementary materials, read and accepted the final version of the manuscript, and gave approval for submission.

## Conflict of interest

The authors declare that the research was conducted in the absence of any commercial or financial relationships that could be construed as a potential conflict of interest.

## Publisher’s note

All claims expressed in this article are solely those of the authors and do not necessarily represent those of their affiliated organizations, or those of the publisher, the editors and the reviewers. Any product that may be evaluated in this article, or claim that may be made by its manufacturer, is not guaranteed or endorsed by the publisher.
