# Supplementary material for: Glycogen synthase kinases in model and crop plants – From negative regulators of brassinosteroid signaling to multifaceted hubs of various signaling pathways and modulators of plant reproduction and yield
Source: Front Plant Sci. 2022 Jul 15;13:939487. doi: 10.3389/fpls.2022.939487 (PMC9335153; doi:10.3389/fpls.2022.939487)
Supplement: Supplementary file 1 [file Data_Sheet_1.PDF]

Supplementary Material

Supplementary Table 1. Nomenclature and classification of representatives of the GSK/SK protein family from various model and crop species.

Species	Acronym	Name	Clade	Gene code	Locus	Database
Arabidopsis thaliana	AtSK11	*Arabidopsis thaliana Shaggy-like Kinase11*	I	P43288-1	At5g26751	UniProt
	AtSK12	*Arabidopsis thaliana Shaggy-like Kinase12*	I	P43289-1	At3g05840	UniProt
	AtSK13	*Arabidopsis thaliana Shaggy-like Kinase13*	I	Q8VZD5-1	At5g14640	UniProt
	AtSK21/BIN2	*Arabidopsis thaliana Shaggy-like Kinase21/ Brassinosteroid-Insensitive2*	II	Q39011-1	At4g18710	UniProt
	AtSK22/BIL2	*Arabidopsis thaliana Shaggy-like Kinase22/ BIN2-like2*	II	Q39012-1	At1g06390	UniProt
	AtSK23/BIL1	*Arabidopsis thaliana Shaggy-like Kinase23/ BIN2-like1*	II	Q39010-1	At2g30980	UniProt
	AtSK31	*Arabidopsis thaliana Shaggy-like Kinase31*	III	O23145	At3g61160	UniProt
	AtSK32	*Arabidopsis thaliana Shaggy-like Kinase32*	III	Q96287-1	At4g00720	UniProt
	AtSK41	*Arabidopsis thaliana Shaggy-like Kinase41*	IV	Q39019-1	At1g09840	UniProt
	AtSK42	*Arabidopsis thaliana Shaggy-like Kinase42*	IV	Q9FVS6-1	At1g57870	UniProt

Oryza sativa	OsSK11	*Oryza sativa Shaggy-like Kinase11*	I		Os01g14860	Rice Genome Annotation Project
	OsSK12	*Oryza sativa Shaggy-like Kinase12*	I		Os01g19150	Rice Genome Annotation Project
	OsSK13	*Oryza sativa Shaggy-like Kinase13*	I		Os05g04340	Rice Genome Annotation Project
	OsSK21	*Oryza sativa Shaggy-like Kinase21*	II		Os01g10840	Rice Genome Annotation Project
	OsSK22	*Oryza sativa Shaggy-like Kinase22*	II		Os05g11730	Rice Genome Annotation Project
	OsSK23	*Oryza sativa Shaggy-like Kinase23*	II		Os02g14130	Rice Genome Annotation Project
	OsSK24	*Oryza sativa Shaggy-like Kinase24*	II		Os06g35530	Rice Genome Annotation Project
	OsSK31	*Oryza sativa Shaggy-like Kinase31*	III		Os10g37740	Rice Genome Annotation Project
	OsSK41	*Oryza sativa Shaggy-like Kinase41*	IV		Os03g62500	Rice Genome Annotation Project
Hordeum vulgare	HvGSK1.1	*Hordeum vulgare Glycogen Synthase Kinase1.1*	I	AK251287.1		NCBI
	HvGSK1.2	*Hordeum vulgare Glycogen Synthase Kinase1.2*	I	AK368391.1		NCBI
	HvGSK1.3	*Hordeum vulgare Glycogen Synthase Kinase1.3*	I	HORVU1Hr1G016490.9		Ensembl Plants

	HvGSK2.1	*Hordeum vulgare Glycogen Synthase Kinase2.1*	II	AK364823.1<br>AK360132.1	NCBI
	HvGSK2.2	*Hordeum vulgare Glycogen Synthase Kinase2.2*	II	MLOC_68311.2	Ensembl Plants
	HvGSK3.1	*Hordeum vulgare Glycogen Synthase Kinase3.1*	III	AK362547.1	NCBI
	HvGSK4.1	*Hordeum vulgare Glycogen Synthase Kinase4.1*	IV	AK358344.1<br>AK360683.1	NCBI
Nicotiana tabacum	NTK-1	*Nicotiana tabacum NtK-1*	I	X77763	NCBI
	NSK6	*Nicotiana tabacum shaggy-like kinase6*	III	Y08607	NCBI
	NSK59	*Nicotiana tabacum shaggy-like kinase59*	III	AJ002315	NCBI
	NSK91	*Nicotiana tabacum shaggy-like kinase91*	III	AJ224163	NCBI
	NSK111	*Nicotiana tabacum shaggy-like kinase111*	III	AJ002314	NCBI
Medicago sativa	MSK-1	*Medicago sativa shaggy-like kinase-1*	I	X68411	NCBI
	MSK-2	*Medicago sativa shaggy-like kinase-2*	I	X68410	NCBI
	MSK-3	*Medicago sativa shaggy-like kinase-3*	I	X68409	NCBI
	WIG	*Wound-induced GSK-3*	III	AJ295939	NCBI
	MSK-4	*Medicago sativa shaggy-like kinase-4*	IV	AF432225	NCBI
Triticum aestivum	TaGSK1	*Triticum aestivum Glycogen Synthase Kinase–shaggy1*	I	AF525086.1	NCBI
	TaSK5	*Triticum aestivum shaggy kinase5*	I	AB281487.1	NCBI
	TaSK1-A	*Triticum aestivum Shaggy-like Kinase1*	II	AGJ93554.1	NCBI

	TaSK1-B	*Triticum aestivum Shaggy-like Kinase1*	II	AGJ93555.1	NCBI
	TaSK1-C	*Triticum aestivum Shaggy-like Kinase1*	II	AGK41247.1	NCBI
	TaSK2-A	*Triticum aestivum Shaggy-like Kinase2*	II	AGK41248.1	NCBI
	TaSK2-B	*Triticum aestivum Shaggy-like Kinase2*	II	AGJ93556.1	NCBI
	TaSK2-C	*Triticum aestivum Shaggy-like Kinase2*	II	AGJ93558.1	NCBI
Brassica rapa		Bra036593	I	Bra036593	BRAD
		Bra009916	I	Bra009916	BRAD
		Bra001169	I	Bra001169	BRAD
		Bra039407	I	Bra039407	BRAD
		Bra040251	I	Bra040251	BRAD
		Bra006263	I	Bra006263	BRAD
		Bra006328	I	Bra006328	BRAD
		Bra021685	II	Bra021685	BRAD
		Bra018285	II	Bra018285	BRAD
		Bra013341	II	Bra013341	BRAD
		Bra020994	II	Bra020994	BRAD
		Bra012581	II	Bra012581	BRAD
		Bra003440	III	Bra003440	BRAD
		Bra007577	III	Bra007577	BRAD
		Bra037354	III	Bra037354	BRAD
		Bra000961	III	Bra000961	BRAD
		Bra019983	IV	Bra019983	BRAD
		Bra030803	IV	Bra030803	BRAD
