# Supplementary material for: Glycogen synthase kinases in model and crop plants – From negative regulators of brassinosteroid signaling to multifaceted hubs of various signaling pathways and modulators of plant reproduction and yield
Source: Front Plant Sci. 2022 Jul 15;13:939487. doi: 10.3389/fpls.2022.939487 (PMC9335153; doi:10.3389/fpls.2022.939487)
Supplement: Supplementary file 2 [file Data_Sheet_2.PDF]

```
OsSK41     --------------------------------------------------MAYSGLRHGG   10
AtSK41     --------------------------------------------------MASSGLGNGV   10
AtSK42     ---------------------------------------------------MESHLGNGV   9
OsSK23     ----------------MATLPGGPHPT-----GGGAA----ADPMQVD------------   23
OsSK24     ----------------MAAMPGGPDLA-----GAGGAVAVAVDAMQVD------------   27
OsSK21     ----------------MEAPP-GPEP------------------MELD------------   13
OsSK22     ---------------MDQPAP-APEP------------------MLLD------------   14
AtSK21     ------------------------------------------------------------   0
AtSK22     ---------------MASLPLGPQP-------------------H-AL------------   13
AtSK23     ---------------MTSIPLGPPQP------------------PSLA------------   15
AtSK31     MNVVRRLTSIASGRNFVSSDNVGETETPRSKPNQNREETESTETT---------------   45
AtSK32     MNVMRRLKSIASGRTSISSDPGGDYALKRAKLDQENDN-LCVDPMQVDQNSSCFEMKADV   59
OsSK31     MHMMRRLKSIASGRSSV-SDPGGDSGTKRPKFDQDGAGDIVIEPHLTDDKPMRVDQESSS   59
OsSK12     --------------------------------------------------MTSVGLVDS-   9
OsSK13     --------------------------------------------------MASVGVVRS-   9
OsSK11     --------------------------------------------------MGSVGVAPS-   9
AtSK13     --------------------------------------------------MASVGTLPA-   9
AtSK11     --------------------------------------------------MASVGIAPN-   9
AtSK12     --------------------------------------------------MASVGIEPS-   9
OsSK41     VG-----SSSRPGHGFKGPASSVECLGREMLEMQLRDSKPDVGDEKNTERDVVDGSSAEA   65
AtSK41     -------GTSRSAKGLKSSSSSVDWLTRDLAETRIRDK-VETDDERDSEPDIIDGAGAEP   62
AtSK42     -------GSSRSAKNTKNTSSSVDWLSRDMLEMKIRDK-TEADEERDSEPDIIDGVGAEP   61
OsSK23     ------------------------QPRSAAA-AAAV-----APAGEKHGASLMEGSDPVT   53
OsSK24     ------------------------DPPR-------------ASAEEKHGPTIMGGNDPVT   50
OsSK21     ------------------------APPPPAAVAAA-AA----TAGISEKKKEEGGGDAVT   44
OsSK22     ------------------------AQPPAAVACDK-KQ----QEG---EAPYAEGNDAVT   42
AtSK21     -----------------------------------------MADDKEMPAAVVDGHDQVT   19
AtSK22     ------------------------APPLQLHDGDALKRRPELDSDKEMSAAVIEGNDAVT   49
AtSK23     ------------------------PQPPHLHGGDSLKRRPDIDNDKEMSAAVIEGNDAVT   51
AtSK31     ----------SYEKDSVSSSENSDHLPKEI--------------REDMDCGIIKGNGTES   81
AtSK32     --LSQESVAGTSNVPAVSEKPVDDQLPDVMIEMKIRDERNANREDKDMETTVVNGSGTET   117
OsSK31     SHRDAEASTSTSKNPGRTEEAGADILPKEMNEMTISDDKVDGHNDKESEGVIVNANGTET   119
OsSK12     ---------SSGFP-ETSTSGATDRLTDDISEMSIRD--------KEVEAVVVSGNSMDI   51
OsSK13     ---------SLGFQNETSTSGDADRLPNEMSNMSIRDD------NKDIDDIVVNGNGTEP   54
OsSK11     ---------GLKN--SSSTSMGAEKLPDQMHDLKIRDD-------KEVEATIINGKGTET   51
AtSK13     ---------SSMATKQSNASICAEKLPEGINEMKIKDD-------KEMEAAVVDGNGTET   53
AtSK11     ---------PGARDS-TG----VDKLPEEMNDMKIRDD-------KEMEATVVDGNGTET   48
AtSK12     ---------AAVRES-TGNVTDADRLPEEMKDMKIQDD-------KEMEATIVNGNVTET   52
OsSK41     GHIIATTIRGRNGLPKQSVTYIAEHVVGTGSFGVVYQAKCRETGEIVAIKKVLQDKRYKN   125
AtSK41     GHVIRTTLRGRNGQSRQTVSYISEHVVGTGSFGMVFQAKCRETGEVVAIKKVLQDKRYKN   122
AtSK42     GHVITTTLPGRNGQSRQTVSYIAEHVVGTGSFGMVFQAKCRETGEVVAIKKVLQDKRYKN   121
OsSK23     GHIISTTIGGKNGEPKRTISYMAERVVGTGSFGIVFQAKCLETGETVAIKKVLQDKRYKN   113
OsSK24     GHIISTTIGGKNDEPKRTISYMAERVVGTGSFGVVFQAKCLETGETVAIKKVLQDKRYKN   110
OsSK21     GHIISTTIGGKNGEPKRTISYMAERVVGTGSFGIVFQAKCLETGETVAIKKVLQDRRYKN   104
OsSK22     GHIISTTIGGKNGEPKRTISYMAERVVGTGSFGIVFQAKCLETGETVAIKKVLQDRRYKN   102
AtSK21     GHIISTTIGGKNGEPKQTISYMAERVVGTGSFGIVFQAKCLETGETVAIKKVLQDRRYKN   79
AtSK22     GHIISTTIGGKNGEPKQTISYMAERVVGTGSFGIVFQAKCLETGESVAIKKVLQDRRYKN   109
AtSK23     GHIISTTIGGKNGEPKQTISYMAERVVGTGSFGIVFQAKCLETGESVAIKKVLQDRRYKN   111
AtSK31     GRIITTKKKGLNDQKDKTISYRAEHVIGTGSFGVVFQAKCLETEEKVAIKKVLQDKRYKN   141
AtSK32     GQVITTTVGGRDGKPKQTISYMAQRVVGTGSFGVVFQAKCLETGEQVAIKKVLQDKRYKN   177
OsSK31     GQIIVTSIGGQNGKPKQKVSYMAERVVGTGSFGVVFQAKCLETGETVAIKKVLQDKRYKN   179
OsSK12     GHTIVTTVGGRNGQPKQTISYIAERAVGRGSFGVVFQAKCLETGERVAVKKVLQDARYKN   111
OsSK13     GHVIVTSIDGRNGQAKQTISYMAERVVGHGSFGTVFQAKCLETGETVAIKKVLQDKRYKN   114
OsSK11     GHIIVTTTGGRNGQPKQTVSYMAERIVGQGSFGIVFQAKCLETGETVAIKKVLQDKRYKN   111
AtSK13     GHIIVTTIGGKNGQPKQTISYMAERIVGQGSFGIVFQAKCLETGETVAIKKVLQDKRYKN   113
AtSK11     GHIIVTTIGGRNGQPKQTISYMAERVVGHGSFGVVFQAKCLETGETVAIKKVLQDRRYKN   108
AtSK12     GHIIVTTIGGRNGQPKQTISYMAERVVGHGSFGVVFQAKCLETGETVAIKKVLQDRRYKN   112
           *: * *.  * :.   :.::* ::: :* **** *:**** ** * **:****** ****
                                                  1
```

```
OsSK41   RELQIMHMLDHPNIVGLKHYFFSTTERDELYLNLVLEYVPETVNRIARQYSRMNQRVPLI   185
AtSK41   RELQIMQMLDHPNAVALKHSFFSRTDNEEVYLNLVLEFVPETVNRVARSYSRTNQLMPLI   182
AtSK42   RELQIMQMLDHPNVVCLKHSFYSRTENEEVYLNLVLEFVPETVNRTARSYSRMNQLMPLI   181
OsSK23   RELQIMRSMDHCNVVSLKHCFFSTTSRDELFLNLVMEFVPESLYRVLKHYSNMNQRMPLI   173
OsSK24   RELQIMRSMDHCNVISLKHCFFSTTSRDELFLNLVMEFVPESLYRVLKHYKDMKQRMPLI   170
OsSK21   RELQLMRAMEHPNVICLKHCFFSTTSRDELFLNLVMEYVPETLYRVLKHYSNANQRMPLI   164
OsSK22   RELQLMRAMDHPNVISLKHCFFSTTSRDELFLNLVMEYVPETLYRVLKHYSNANHRMPLI   162
AtSK21   RELQLMRVMDHPNVVCLKHCFFSTTSKDELFLNLVMEYVPESLYRVLKHYSSANQRMPLV   139
AtSK22   RELQLMRPMDHPNVISLKHCFFSTTSRDELFLNLVMEYVPETLYRVLRHYTSSNQRMPIF   169
AtSK23   RELQLMRLMDHPNVVSLKHCFFSTTTRDELFLNLVMEYVPETLYRVLKHYTSSNQRMPIF   171
AtSK31   RELQIMRMLDHPNVVELKHSFFSTTEKDELYLNLVLEYVPETIYRASRSYTKMNQHMPLI   201
AtSK32   RELQIMRLQDHPNVVRLRHSFFSTTDKDELYLNLVLEYVPETVYRASKHYTKMNQHMPII   237
OsSK31   RELQTMQLLDHPNVVQLKHHFFSTTERGEVYLNLVLEYVSETVYRVAKYYNRMNQRVPIL   239
OsSK12   RELQTMQVLDHPNVACLKHYFCSTTAKEELYLNLVLEYVPETVHRVIRHYNKMSQRMPLI   171
OsSK13   RELQTMRVLDHPNVVSLKHCFFSKTEKEELYLNLVLEYVPETAHRVIKHYNKMNQRMPLI   174
OsSK11   RELQTMRLLDHPNVVALKHCFFSTTEKDELYLNLVLEYVPETVHRVVKHYNKMNQRMPLI   171
AtSK13   RELQTMRLLDHPNVVSLKHCFFSTTEKDELYLNLVLEYVPETVYRVSKHYSRANQRMPII   173
AtSK11   RELQTMRLLDHPNVVSLKHCFFSTTEKDELYLNLVLEYVPETVHRVIKHYNKLNQRMPLI   168
AtSK12   RELQTMRLLDHPNVVSLKHCFFSTTEKDELYLNLVLEYVPETVHRVIKHYNKLNQRMPLV   172
         **** *:  :* *   *:* * * * . *::****:*:* *:  *  : *.  .: :*:.
                        2   3

OsSK41   YVKLYTYQICRALAYIHNCVGICHRDIKPQNVLVNPHTHQLKICDFGSAKVLVKGEPNIS   245
AtSK41   YVKLYTYQICRALAYIHNSFGLCHRDIKPQNLLVNPHTHQLKICDFGSAKVLVKGEPNVS   242
AtSK42   YVKLYTYQICRGLAYLHNCCGLCHRDIKPQNLLVNPHTHQLKICDFGSAKVLVKGEPNIS   241
OsSK23   YVKLYVYQIFRGLAYIHTVPGVCHRDVKPQNLLVDPLTHQVKICDFGSAKMLVKGEANIS   233
OsSK24   YVKLYMYQIFRGLAYIHTVPGVCHRDIKPQNILVDPLTHQVKVCDFGSAKMLIKGEANIS   230
OsSK21   YVKLYIYQLFRGLAYIHTVPGVCHRDVKPQNVLVDPLTHQVKLCDFGSAKVLVPGEPNIS   224
OsSK22   YVKLYMYQLFRGLAYIHTVPGVCHRDVKPQNVLVDPLTHQVKLCDFGSAKTLVPGEPNIS   222
AtSK21   YVKLYMYQIFRGLAYIHNVAGVCHRDLKPQNLLVDPLTHQVKICDFGSAKQLVKGEANIS   199
AtSK22   YVKLYTYQIFRGLAYIHTVPGVCHRDVKPQNLLVDPLTHQVKLCDFGSAKVLVKGEPNIS   229
AtSK23   YVKLYTYQIFRGLAYIHTAPGVCHRDVKPQNLLVDPLTHQCKLCDFGSAKVLVKGEANIS   231
AtSK31   YIQLYTYQICRAMNYLHQVVGVCHRDIKPQNLLVNNVTHEVKICDFGSAKMLIPGEPNIS   261
AtSK32   FVQLYTYQICRALNYLHRVVGVCHRDIKPQNLLVNPQTHQLKICDFGSAKMLVPGEPNIS   297
OsSK31   HVKLYAYQMCRALAYIHRVVGVCHRDIKPQNLLVNPHTHQLKLCDFGSAKKLVPGEPNIS   299
OsSK12   YVKLYMYQICRALAYIHNCVGVCHRDIKPQNILVNPHNHQLKLCDFGSAKVLVKGEPNIS   231
OsSK13   YAKLYMYQICRALAYIHNTIGVCHRDIKPQNLLVNPHTHQLKLCDFGSAKVLVKGEPNIS   234
OsSK11   YVKLYMYQICRALAYIHNSIGVCHRDIKPQNLLVNPHTHQLKLCDFGSAKVLVKGEPNIS   231
AtSK13   YVKLYTYQICRALAYIHGGVGVCHRDIKPQNLLVNPHTHQVKLCDFGSAKVLVKGEPNIS   233
AtSK11   YVKLYTYQIFRALSYIHRCIGVCHRDIKPQNLLVNPHTHQVKLCDFGSAKVLVKGEPNIS   228
AtSK12   YVKLYTYQIFRSLSYIHRCIGVCHRDIKPQNLLVNPHTHQVKLCDFGSAKVLVKGEPNIS   232
         . :** **: *.: *:*   *:****:****:**:  .*: *:******* *: ** *:*
                               4                        5 6

OsSK41   YICSRYYRAPELIFGATEYTTAIDLWSTGCVMAELLLGQPLFPGESGVDQLVEIIKVLGT   305
AtSK41   YICSRYYRAPELIFGASEYTTAIDIWSTGCVMAELLLGQPLFPGESGVDQLVEIIKVLGT   302
AtSK42   YICSRYYRAPELIFGATEYTTAIDIWSTGCVMAELLLGQPLFPGESGVDQLVEIIKVLGT   301
OsSK23   YICSRYYRAPELIFGATEYTTSIDIWSAGCVLAELLLGQPLFPGESAVDQLVEIIKVLGT   293
OsSK24   YICSRYYRAPELIFGATEYTTSIDIWSAGCVLAELLLGQPLFPGESAVDQLVEIIKVLGT   290
OsSK21   YICSRYYRAPELIFGATEYTTSIDIWSAGCVLAELLLGQPLFPGESAVDQLVEIIKVLGT   284
OsSK22   YICSRYYRAPELIFGATEYTTSIDIWSAGCVLAELLLGQPLFPGESAVDQLVEIIKVLGT   282
AtSK21   YICSRFYRAPELIFGATEYTTSIDIWSAGCVLAELLLGQPLFPGENAVDQLVEIIKVLGT   259
AtSK22   YICSRYYRAPELIFGATEYTASIDIWSAGCVLAELLLGQPLFPGENSVDQLVEIIKVLGT   289
AtSK23   YICSRYYRAPELIFGATEYTSSIDIWSAGCVLAELLLGQPLFPGENSVDQLVEIIKVLGT   291
AtSK31   YICSRYYRAPELIFGATEYTSAIDMWSVGCVMAELFLGHPLFPGETSVDQLVEIIKILGT   321
AtSK32   YICSRYYRAPELIFGATEYTNAIDMWSGGCVMAELLLGQPLFPGESGIDQLVEIIKILGT   357
OsSK31   YICSRYYRAPELIFGATEYTTAIDIWSVGCVLAELLIGQPLFPGESGVDQLVEIIKILGT   359
OsSK12   YICSRYYRAPELIFGATEYTTAIDVWSAGCVLAELLLGQPVFPGDSGVDQLVEIIKVLGT   291
OsSK13   YICSRYYRAPELIFGATEYTTAIDVWSAGCVLAELLLGQPLFPGDSGVDQLVEIIKVLGT   294
OsSK11   YICSRYYRAPELIFGATEYTTAIDIWSAGCVLAELMLGQPLFPGESGVDQLVEIIKVLGT   291
AtSK13   YICSRYYRAPELIFGATEYTTTIDIWSAGCVLAELLLGQPLFPGESGVDQLVEIIKVLGT   293
AtSK11   YICSRYYRAPELIFGATEYTTAIDVWSAGCVLAELLLGQPLFPGESGVDQLVEIIKVLGT   288
AtSK12   YICSRYYRAPELIFGATEYTTAIDVWSAGCVLAELLLGQPLFPGESGVDQLVEIIKVLGT   292
         *****:**********:*** :**:** ***:***::*:*:***:..:********:***
         7  8
```

```
OsSK41    PTREEIKCMNPNYTEFKFPQIKAHPWHKVFQKRLPPEAVDLVSRFLQYSPNLRCTAM---   362
AtSK41    PTREEIKCMNPNYTEFKFPQIKPHPWHKVFQKRLPPEAVDLLCRFFQYSPNLRCTAL---   359
AtSK42    PTREEIKCMNPNYTEFKFPQIKPHPWHKVFQKRLPPEAVDLLCRFFQYSPNLRCTAV---   358
OsSK23    PTREEIRCMNPNYTEFRFPQIKAHPWHKIFHKRMPPEAIDLASRLLQYAPNLRCTAL---   350
OsSK24    PTREEIRCMNPNYTEFKFPQIKACPWHKIFHKRMPPEAIDLVSRLLQYSPNLRCTAL---   347
OsSK21    PTREEIRCMNPNYTEFKFPQIKAHPWHKIFHKRMPPEAIDLASRLLQYSPSLRCTAL---   341
OsSK22    PTREEIRCMNPNYTEFRFPQIKAHPWHKVFHKRMPPEAIDLASRLLQYSPSLRCTAL---   339
AtSK21    PTREEIRCMNPHYTDFRFPQIKAHPWHKIFHKRMPPEAIDFASRLLQYSPSLRCTAL---   316
AtSK22    PTREEIRCMNPNYTDFRFPQIKAHPWHKVFHKRMPPEAIDLASRLLQYSPSLRCTAL---   346
AtSK23    PTREEIRCMNPNYTDFRFPQIKAHPWHKVFHKRMPPEAIDLASRLLQYSPSLRCTAL---   348
AtSK31    PAREEIKNMNPRYNDFKFPQIKAQPWHKIFRRQVSPEAMDLASRLLQYSPNLRCTAL---   378
AtSK32    PTREEIRCMNPNYTEFKFPQIKAHPWHKIFHKRMPPEAVDLVSRLLQYSPNLRCTAL---   414
OsSK31    PTREEIRCMNPNYSEFKFPQIKAHPWHKLFGKRMPPEAVDLVSRLLQYSPNLRCTAV---   416
OsSK12    PTREEIKHMNPNYTEFKFPQIKAHPWHKIFHKRMPSEAVDLVSRLLQYSPHLRCSAVSIN   351
OsSK13    PTREEIKCMNPNYTEFKFPQIKAHPWHKIFHKRMPAEAVDLVSRLLQYSPYLRSTAS---   351
OsSK11    PTREEIKCMNPNYTEFKFPQIKAHPWHKVFHKRLPPEAVDLVSRLLQYSPNLRCTAV---   348
AtSK13    PTREEIKCMNPNYTEFKFPQIKAHPWHKIFHKRTPPEAVDLVSRLLQYSPNLRSTAM---   350
AtSK11    PTREEIKCMNPNYTEFKFPQIKAHPWHKIFHKRMPPEAVDLVSRLLQYSPNLRSAAL---   345
AtSK12    PTREEIKCMNPNYTEFKFPQIKAHPWHKIFHKRMPPEAVDLVSRLLQYSPNLRCAAL---   349
          *:****: ***.*.:*:*****  ****:* ::   **:*: .*::**:* **.:*
            9

OsSK41    --------------------------EACMHPFFDELRDPNTRLPNGRPLPPLFNFRTQE   396
AtSK41    --------------------------EACIHPLFDELRDPNTRLPNGRPLPPLFNFKPQE   393
AtSK42    --------------------------EACIHPFFDELRDPNARLPNGRPLPPLFNFKPQE   392
OsSK23    --------------------------EACAHSFFDELREPHARLPNGRPFPPLFNFK-QE   383
OsSK24    --------------------------EACAHSFFDELREPHAKLPNGRPFPPLFNFK-QE   380
OsSK21    --------------------------DACAHSFFDELREPNARLPNGRPFPPLFNFK-HE   374
OsSK22    --------------------------DACAHPFFDELREPNARLPNGRPFPPLFNFK-HE   372
AtSK21    --------------------------EACAHPFFDELREPNARLPNGRPFPPLFNFK-QE   349
AtSK22    --------------------------EACAHPFFNELREPNARLPNGRPLPPLFNFK-QE   379
AtSK23    --------------------------EACAHPFFNELREPNARLPNGRPLPPLFNFK-QE   381
AtSK31    --------------------------EACAHPFFDDLRDPRASLPNGRALPPLFDFTAQE   412
AtSK32    --------------------------EACAHPFFDDLRDPNVSLPNGRALPPLFNFTAQE   448
OsSK31    --------------------------DACAHPFFDELRDPKTCLSNGRSLPPLFDFSAAE   450
OsSK12    AYIRKSLVNVSMYSFYIFLFFSSHQLEVLIHPFFDELRDPNARLPNGRTLPPLFNFKPRE   411
OsSK13    --------------------------EALIHPFFDELRDPNTRLPNGRFLPPLFNFKPHE   385
OsSK11    --------------------------EALVHPFFDELRDPNARLPNGRFLPPLFNFKPHE   382
AtSK13    --------------------------EAIVHPFFDELRDPNTRLPNGRALPPLFNFKPQE   384
AtSK11    --------------------------DTLVHPFFDELRDPNARLPNGRFLPPLFNFKPHE   379
AtSK12    --------------------------DSLVHPFFDELRDPNARLPNGRFLPPLFNFKPHE   383
                                    :   * :*::**:*.. * *** :****:*   *

OsSK41    LNGIPPEAIERLVPEHARRQSLFMALRT---   424
AtSK41    LSGIPPEIVNRLVPEHARKQNLFMALHS---   421
AtSK42    LSGIPPETVDRLVPEHARKQNHFMALHS---   420
OsSK23    LANLSPELINRLIPEHAR-------------   401
OsSK24    LANTHPELVSRLLPEHAQRHSGF--------   403
OsSK21    LASASPELIHRLIPDHIRRQHGLNFAHAGS-   404
OsSK22    LANSSQELISRLIPEHVRRQATHNFFNTGS-   402
AtSK21    VAGSSPELVNKLIPDHIKRQLGLSFLNQSGT   380
AtSK22    LGGASMELINRLIPEHVRRQMSTGLQNS---   407
AtSK23    LSGASPELINRLIPEHVRRQMNGGFPFQAGP   412
AtSK31    LAGASVELRHRLIPEHARK------------   431
AtSK32    LAGASTELRQRLIPAHCQGTGSSS-------   472
OsSK31    LEGLPVELVHRIIPEHMRK------------   469
OsSK12    LKGASMEFLVKLVPQHAKKQCAFLGL-----   437
OsSK13    LKGMPMEFLVKLIPEHARKQCAFVGW-----   411
OsSK11    LKGIPSDIMAKLIPEHVKKQCSYAGV-----   408
AtSK13    LKGASLELLSKLIPDHARKQCSFLAL-----   410
AtSK11    LKGVPLEMVAKLVPEHARKQCPWLGL-----   405
AtSK12    LKGVPVEMVAKLVPEHARKQCPWLSL-----   409
          : .   :   :::* *  :
```

Supplementary Figure 1. Alignment of the SK/GSK protein sequences from Arabidopsis and rice. Accession numbers of the sequences and databases from which they were retrieved are given in the Supplementary Table 1. Positions of the amino-acid residues which are modified during regulation of the AtSK21/BIN2 protein activity are shown in black frames: 1 - Cys^{59}, 2- Cys^{95}, 3 - Cys^{99}, 4 - Cys^{162}, 5 - Ser^{187}, 6 - Lys^{189}, 7 - Tyr^{200}, 8 - Ser^{203}, 9 - Thr^{261}, Arg^{262}, Glu^{263}, Glu^{264} (TREE motif). The alignment was prepared using the Clustal Omega program.
